# An enhanced genetic model of colorectal cancer progression history

**DOI:** 10.1186/s13059-019-1782-4

**Published:** 2019-08-15

**Authors:** Lixing Yang, Su Wang, Jake June-Koo Lee, Semin Lee, Eunjung Lee, Eve Shinbrot, David A. Wheeler, Raju Kucherlapati, Peter J. Park

**Affiliations:** 1 Ben May Department for Cancer Research and Department of Human Genetics, The University of Chicago, Chicago, IL USA; 2 Department of Biomedical Informatics, Harvard Medical School, Boston, MA USA; 3 Ludwig Center at Harvard, Boston, MA USA; 4 Department of Molecular and Human Genetics, Baylor College of Medicine, Houston, TX USA; 5 Division of Genetics, Brigham and Women’s Hospital, Boston, MA USA; 6 Department of Genetics, Harvard Medical School, Boston, MA USA; 7 Present Address: Department of Biomedical Engineering, Ulsan National Institute of Science and Technology, Ulsan, South Korea; 8 Present Address: Division of Genetics and Genomics, Boston Children’s Hospital, Boston, MA USA

**Keywords:** Tumor evolution, Tumor heterogeneity, Aneuploidy, Kataegis

## Abstract

**Background:**

The classical genetic model of colorectal cancer presents *APC* mutations as the earliest genomic alterations, followed by *KRAS* and *TP53* mutations. However, the timing and relative order of clonal expansion and other types of genomic alterations, such as genomic rearrangements, are still unclear.

**Results:**

Here, we perform comprehensive bioinformatic analysis to dissect the relative timing of somatic genetic alterations in 63 colorectal cancers with whole-genome sequencing data. Utilizing allele fractions of somatic single nucleotide variants as molecular clocks while accounting for the presence of copy number changes and structural alterations, we identify key events in the evolution of colorectal tumors. We find that driver point mutations, gene fusions, and arm-level copy losses typically arise early in tumorigenesis; different mechanisms act on distinct genomic regions to drive DNA copy changes; and chromothripsis—clustered rearrangements previously thought to occur as a single catastrophic event—is frequent and may occur multiple times independently in the same tumor through different mechanisms. Furthermore, our computational approach reveals that, in contrast to recent studies, selection is often present on subclones and that multiple evolutionary models can operate in a single tumor at different stages.

**Conclusion:**

Combining these results, we present a refined tumor progression model which significantly expands our understanding of the tumorigenic process of human colorectal cancer.

**Electronic supplementary material:**

The online version of this article (10.1186/s13059-019-1782-4) contains supplementary material, which is available to authorized users.

## Background

Somatic mutations in colorectal cancer, the third most common cancer worldwide, have been characterized extensively over the years. These include frequent mutations in *TP53*, *APC*, and *KRAS* and are associated with disruptions to pathways such as Wnt signaling, TGF-β signaling, and DNA mismatch repair [1]. In terms of genome stability, microsatellite instability (MSI) and chromosomal instability (CIN) are the two prominent features. MSI cases have deficient DNA mismatch repair due to mutations or epigenetic silencing of mismatch repair genes, such as *MSH2* and *MLH1*; this deficiency results in a multitude of somatic single nucleotide variants (SNVs) and small indels. In contrast, tumors characterized by CIN usually are aneuploid and accumulate somatic structural variants (SVs; also referred to as genomic rearrangements) that frequently produce corresponding copy number variations (CNVs).

Among the many genomic profiling efforts of colorectal cancers, one of the most comprehensive ones was performed by The Cancer Genome Atlas (TCGA), which analyzed nearly 300 cases utilizing multiple “omics” platforms [2]. This analysis identified a subset of ultra-mutated (more mutations than hyper-mutated MSI tumors) tumors carrying mutations in the *POLE* (DNA polymerase epsilon) exonuclease domain, additional frequently mutated genes such as *ARID1A* and *SOX9*, recurrent amplifications of *ERBB2* and *IGF2*, recurrent fusions of *NAV2*-*TCF7L1*, and the importance of transcription regulation of *MYC*. Other studies uncovered various SV drivers for colorectal cancers, including *VTI1A*-*TCF7L2* fusions [3], *RSPO* fusions [4], and somatic transposable element insertions [5].

Although genetic alterations in colorectal cancer have been extensively studied, less is known about the timing of the alterations during tumor progression. The classical genetic model posits the order of major drivers to be *APC*, *TGF* beta, *KRAS*, and *TP53*. The relative timing of these mutations is derived from mouse models [6, 7] and observed mutational frequencies at different tumor stages [8], e.g., *APC* mutations are most frequent in early-stage adenomas and *TP53* mutations become frequent only in late-stage carcinomas. To define the timing of a larger set of alterations, one strategy is to compare mutational frequencies in multiple samples from the same patient [9–12], assuming that the alterations found in all biopsies of a patient must have occurred before the alterations found only in a subset.

Interestingly, a recent study that profiled many individual tumor glands from each colorectal cancer patient found subclonal alterations present in distant regions, suggesting that the majority of subclonal alterations occurred earlier, before ancestral cells expanded in different directions [13]. The authors proposed a “Big Bang” model, where tumors grow as a single expansion without selective sweep. In the absence of multiple samples from an individual, it is also possible to infer relative timing by analyzing allelic fractions in a single bulk sample, using allelic fractions to differentiate subclonal alterations from clonal ones. Another recent study modeling subclonal mutations in TCGA genomes and exomes, including colorectal cancer, concluded again that tumor growth is neutral after initial clonal expansion [14]. The analysis we describe in this paper disputes these inferences.

In this study, we used previously unpublished whole-genome sequencing data from 63 TCGA colorectal cancer patients to deepen our understanding of the evolution of colorectal tumors and the mutational mechanisms that shape their history. In particular, we integrated information from multiple types of genomic alterations to infer the order of mutational events. The events we characterized include somatic SNVs, aneuploidy, genomic rearrangements, clonal expansion, and other complex events such as chromothripsis and kataegis. The use of whole-genome data is essential for our analysis, as it enables more accurate characterization of DNA copy number, mutant allelic fractions, and other genomic features.

## Results

### The landscape of genetic alterations in colorectal cancer

A total of 65 colorectal cancer patients were subjected to whole-genome sequencing (WGS) by TCGA Network at an average depth of 55× for tumor samples and 36× for matched normal samples (Additional file 1: Table S1 and S2). Two samples were excluded from further analysis due to the presence of a large number of small tandem duplications, whose even distribution across the genome indicated amplification-related artifacts (Additional file 2: Figure S1a). Among the 63 high-quality cases, we detected a median of 20,867 somatic SNVs, 4020 small indels, 45 SVs, and 18 transposable element insertions (TEIs) per tumor.

**Fig. 1:**
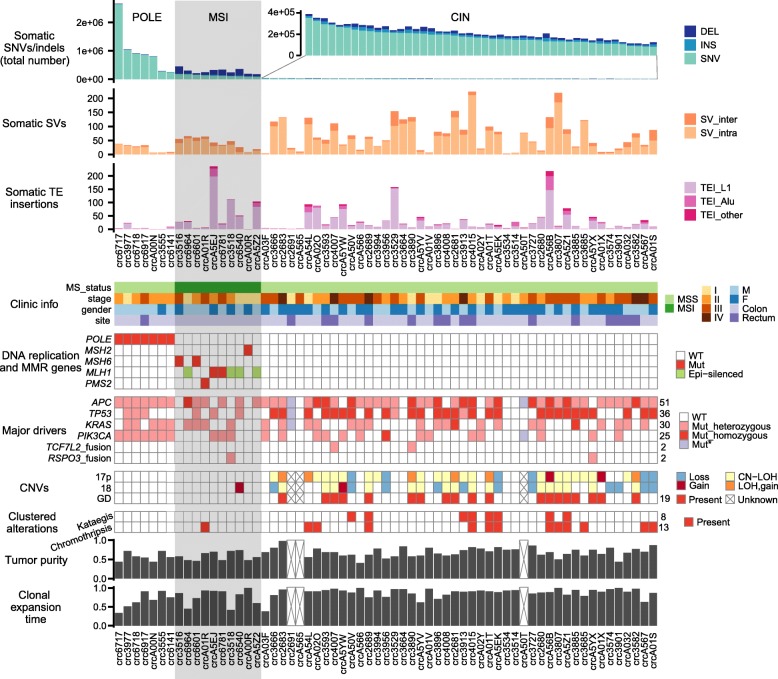
Mutational landscape of our colorectal cancer cohort. Tumors are classified into three groups: *POLE*-mutant, MSI, and CIN. The total number of tumors carrying specific mutations is listed on the right side of the mutation tables. Mut_homozygous indicates all chromosomes in tumor cells carry the mutation regardless of copy number, Mut_heterozygous indicates some chromosomes carry mutant allele and some carry wild-type allele, and Mut* indicates mutations are detected but we cannot determine if some or all chromosomes carry the mutations

The patterns of somatic alterations (Fig. 1) were largely consistent with a previous study [2]. Ten tumors (16%) had mismatch repair (MMR) genes disrupted by either somatic SNVs or epigenetic silencing and displayed the classical MSI phenotype (hyper-mutated and abundant in small indels). Another seven (11%) tumors had a *POLE* exonuclease domain defect, which resulted in ultra-mutated genomes with mutation rates higher than those in MSI tumors [15]. The remaining 46 tumors carried few SNVs and indels but were abundant in CNVs and SVs, consistent with typical characteristics of tumors with CIN. Using the method we developed previously for TEI detection [5], we confirmed that MSI tumors had significantly more somatic TEIs than other tumors (*P* = 0.050 for MSI vs. *POLE*-mutant and CIN tumors combined, Wilcoxon rank sum test), whereas *POLE*-mutant and CIN tumors were not significantly different (*P* = 0.117, Wilcoxon rank sum test).

In our cohort, 6% of tumors had driver fusions. We detected a *VTI1A*-*TCF7L2* fusion and two *RSPO3* fusions. We also found an in-frame *ACSL5*-*TCF7L2* fusion that is part of a complex rearrangement resulting in three distinct in-frame fusions (Additional file 2: Figure S1b). The *ACSL5*-*TCF7L2* fusion activates the 3′ fusion partner *TCF7L2* (Additional file 2: Figure S1c) and therefore is likely to be a driver fusion [16].

### Mutational history revealed by somatic SNVs

**Fig. 2:**
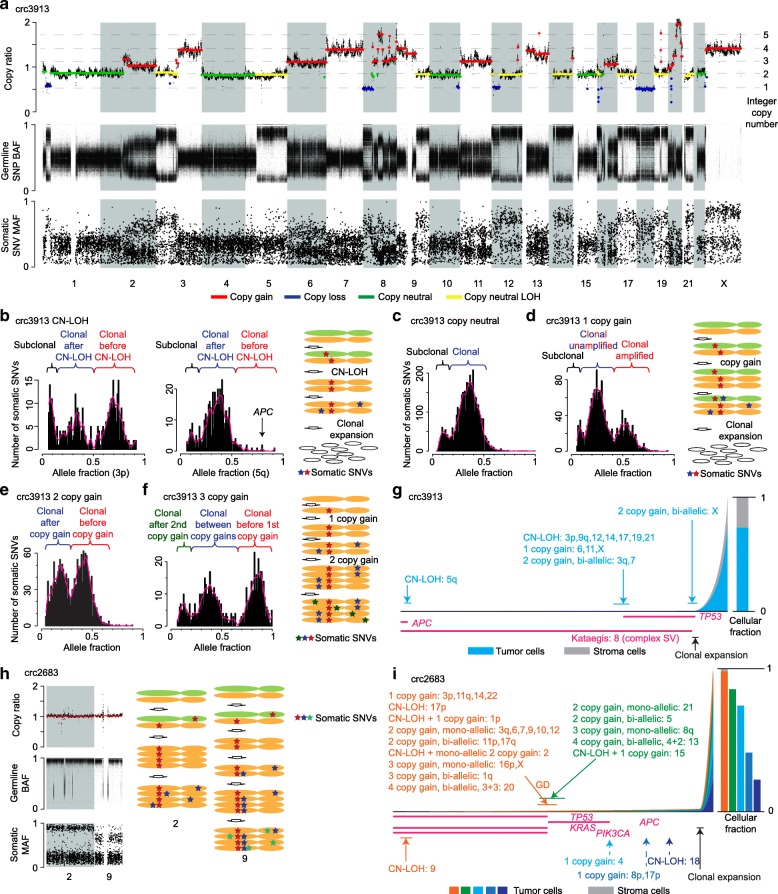
Timing of genetic alterations and tumor progression history. **a** Genetic alteration profiles for tumor crc3913. Copy ratio, heterozygous SNP BAF, and somatic SNV MAF profiles are displayed. The numbers on the right side of the copy ratio plot represent predicted integer copy numbers. **b** MAF profiles and timing estimation for CN-LOH regions. The two plots on the left are allele fraction distributions in two CN-LOH regions, and the plots on the right demonstrate how somatic SNVs accumulate in a CN-LOH chromosome. **c–f** MAF profiles for copy-neutral, one-copy gain, bi-allelic two-copy gain, and three-copy gain chromosomes. Mutational history is shown for one-copy gain and three-copy gain chromosomes. In one-copy gain chromosomes (panel **d**), the SNVs in the middle peak of the MAF profile are SNVs present on one out of the three chromosomes and are a mixture of SNVs occurring both before (red SNVs) and after (blue SNVs) the copy gain, since red SNVs on the green chromosome are not duplicated. **g** Tumor progression map of crc3913. The tumor progresses from left to right. The heights of colored bars on the right represent the proportions of cells. For example, in this tumor, 74% of the cells are tumor cells and 26% of cells are stroma cells. The arrows point out the timing of the events with the 95% of confidence interval shown as a bar above or below the arrows. The time windows when point mutations occur are shown as pink horizontal lines. **h** Complex aneuploidy history inferred by data integration for chromosomes 2 and 9. The variants’ profiles are shown on the left and the proposed mutational history is on the right. **i** Tumor progression map of crc2683

To reconstruct the mutational history of a tumor, we first dissected clonal and subclonal structure and determined ploidy for all chromosomes. Existing methods combine CNV calls with either somatic SNV [17] or germline single nucleotide polymorphism (SNP) calls [18, 19] to infer tumor purity, presence of subclone(s), and integer copy number of each chromosome (equivalent to ploidy) from bulk sequencing data. We integrated *both* mutant allele fractions (MAFs) of somatic SNVs and minor allele (B-allele) frequencies (BAFs) of heterozygous SNPs with copy ratios between tumor and normal samples to infer tumor purity, ploidy, and subclone structure (see the “Material and methods” section). This integrative analysis was challenging due to the high noise level of the various mutational profiles, as illustrated in Fig. 2a. Compared to the data generated by SNP-based platforms, WGS data provide much more accurate inference of copy number profiles, which serve as a basis for purity and ploidy estimation as well as subsequent analysis. Although computational methods addressing this issue have improved substantially [20, 21], because of the lack of sufficient precision in allele fraction quantification and the inherent complexity of the mutational event space, manual annotation is required to properly combine multiple data sources, especially for subclonal inferences. We were able to reconstruct the main events in the history of nearly all 63 cases, performing a manual inspection of each event for accuracy.

To illustrate our procedure of validating the predicted purity and ploidy with somatic SNVs, consider the colorectal sample (crc3913) highlighted in Fig. 2a. The sample’s tumor purity was predicted to be 0.74. Both chromosome arms 3p and 5q were characterized by copy neutral loss of heterozygosity (CN-LOH) based on their copy number of two and the presence of a single allele inferred from the germline BAF distribution. In terms of somatic SNVs, there were three distinct groups according to their allele fractions in both the 3p and 5q (Fig. 2b) arms: clonal SNVs that occurred before the CN-LOH, clonal SNVs that occurred after the CN-LOH, and subclonal SNVs. The somatic SNVs found on both copies of the chromosomes (red SNVs) were predicted to have a MAF of 0.74 based on the formula described in the “Materials and methods” section. The right-most peak was observed at 0.7 for chromosome 3p (Fig. 2b, left panel), close to the predicted value. Similarly, the predicted and observed right-most peaks were 0.37 and 0.37 for copy neutral chromosomes (i.e., chromosomes 1, 2p, 3, 5p, 10, 15, and 22; Fig. 2c), 0.54 and 0.5 for chromosomes (chromosomes 6 and 11) with a one-copy gain (Fig. 2d), and 0.43 and 0.44 for chromosomes (chromosomes 3q, 7 and 13) with a bi-allelic two-copy gain (Fig. 2e), respectively. Interestingly, chromosome X had two separate duplications—a one-copy gain and a bi-allelic two-copy gain based on the somatic MAF pattern (Fig. 2f). The red SNVs occurred before any copy gains and thus were present on all four copies of the chromosome; the blue SNVs occurred after the one-copy gain but before the two-copy gain; and the green SNVs occurred after the two-copy gain (Fig. 2f). The predicted and observed peak positions were 0.85 and 0.88 for red SNVs, respectively. Overall, this suggests the observed MAFs were consistent with predicted tumor purity and ploidy.

To estimate the timing of chromosomal duplications and clonal expansions, we used somatic SNVs as a molecular clock. For example, for the CN-LOH chromosomes of crc3913, there were very few pre-LOH somatic SNVs (red SNVs) on 5q, whereas there were many on 3p, with more post-LOH SNVs (blue SNVs) than pre-LOH ones (Fig. 2b). This suggested that the 5q CN-LOH was a very early event and the 3p CN-LOH happened at a later time. The timing of the CN-LOH could be quantified as 0.02 and 0.68 for 5q and 3p, respectively, based on the number of SNVs occurring before and after the CN-LOH (Fig. 2g). Early CN-LOH events were also present in some other tumors, whereas the rest of the copy gain events usually took place after *TP53* mutations (Additional file 2: Figure S2). This is expected because although cells with wild-type *TP53* cannot tolerate aneuploidy, they can tolerate CN-LOH, as the total number of chromosomes remains the same in CN-LOH regions. Similarly, the timing of the one-copy gain and the bi-allelic two-copy gain could be estimated as 0.66 and 0.71, respectively (Fig. 2d, e) and were very close to the timing of most CN-LOH (Fig. 2g). The timing of clonal expansion was estimated by counting the number of clonal SNVs out of total somatic SNVs. In crc3913, it was estimated to be 0.9 on both CN-LOH (Fig. 2b) and copy neutral non-LOH chromosomes (Fig. 2c). In our study, we only used regions with one or two copies for clonal expansion time estimation because the subclonal peaks of somatic SNVs in MAF profiles were better separated from clonal ones. The inference of clonal expansion time was less reliable on higher copy number chromosomes since subclonal SNVs were often undetectable (Fig. 2e, f). We could also infer when somatic SNVs arose based on their MAFs. For instance, the *APC* mutation in the colorectal tumor crc3913 clearly occurred before the 5q CN-LOH because it was present on both copies of chromosome 5q (Fig. 2b, g). The *TP53* mutation, in contrast, was only present on one copy of chromosome 17 and must have occurred after the CN-LOH (Fig. 2g). The timing estimations of point mutations often encompassed wide windows (the entire pink block in Fig. 2g) since we could only determine the point mutation occurrences to be before or after copy changes/clonal expansions.

In some cases, it was possible to reconstruct complex aneuploidy history. In tumor crc2683, for instance, chromosomes 2 and 9 had the same copy ratios and heterozygous SNP profiles, but they differed in somatic SNV distributions (Fig. 2h). Based on SNPs and copy ratio, we concluded there were four copies of both chromosomes and all four were of the same parental origin. A significant portion of somatic SNVs on chromosome 2 had allele fractions near 1 while many SNVs on chromosome 9 had allele fractions around 0.75 (Fig. 2h and Additional file 2: Figure S3a). The simplest model compatible with these observations is that chromosome 2 had a three-copy gain after losing one parental copy, while chromosome 9 had an early CN-LOH event followed by a late mono-allelic two-copy gain (Fig. 2h, i). We compared our timing estimations with the cancerTiming package [22] in CN-LOH, one-copy gain and mono-allelic two-copy gain regions of all CIN tumors. The two estimations were very similar (*R*^2^ = 0.83). However, the cancerTiming algorithm can only model a subset of evolutionary histories. It cannot model bi-allelic two-copy gain and some complex histories, e.g., two types of copy-gain LOH events in Fig. 2h. In summary, our approach can provide a valuable view of tumor progression history using bulk sequencing data.

**Fig. 3:**
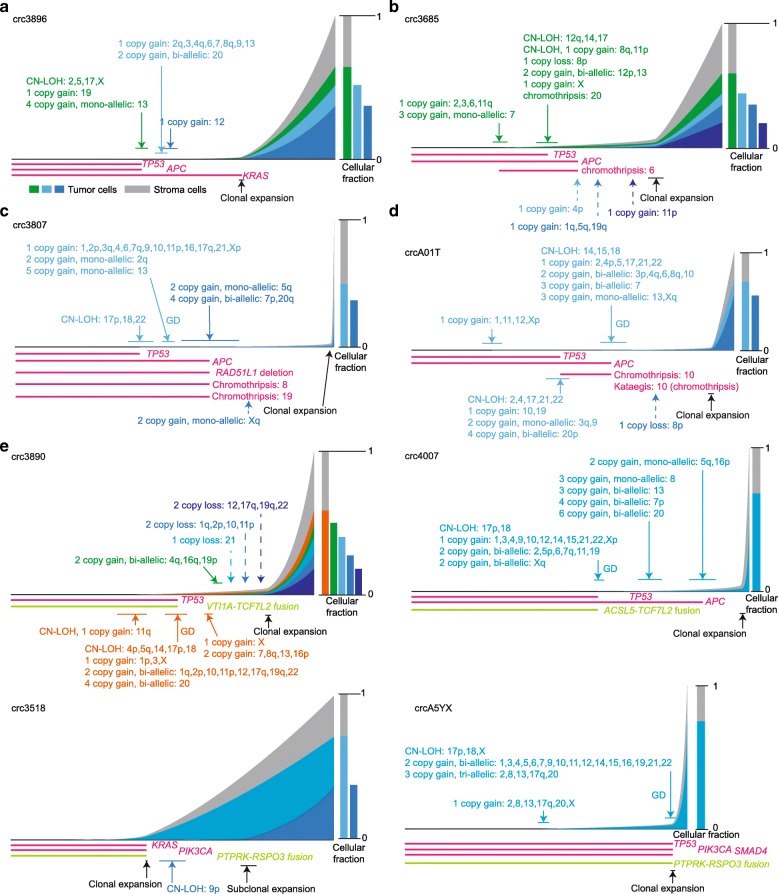
Timing of subclonal CNVs, driver SNVs, and driver gene fusions. **a** Tumor progression map of crc3896. Subclonal copy changes occur before the major clonal expansion. **b–d** Tumor progression maps of crc3685, crc3807, and crcA01T. Driver SNVs often occur early and before aneuploidy. **e** Timing of driver fusions. Three out of four driver fusions occur quite early

We were able to reconstruct the tumor progression history for the vast majority of CIN tumors (43 out of 46; Additional file 2: Figure S4a) as well as all of the MSI and *POLE*-mutant tumors (Additional file 2: Figure S4b and S4c). Interestingly, tumor purity was significantly higher in the CIN tumors than *POLE*-mutant and MSI tumors (*P* = 0.002, Wilcoxon rank sum test; Additional file 2: Figure S4d). In addition, we found that clonal expansion happened earlier for *POLE*-mutant tumors than CIN and MSI tumors (*P* = 0.019, Wilcoxon rank sum test; Additional file 2: Figure S4d). The underlying biological mechanisms of these differences are unknown, but they are unlikely to be caused by mutation burden or mutation mechanisms since our timing estimation of clonal expansion is based on the ratio between clonal and subclonal SNVs. Comparing the timing of subclonal events and clonal expansion, we showed that subclonal copy changes often arise before major clonal expansion (Fig. 3a and Additional file 2: Figure S5). This is consistent with the observation of a previous multi-sample sequencing study [13], but our approach to model the timing of subclonal alterations using a single tumor sample is substantially less burdensome in practice than sequencing multiple samples. We found that driver SNVs often occur before aneuploidy and that mutant alleles were amplified by copy gains (Fig. 3b–d). Similarly, driver fusions also occurred fairly early. This was the case in three of the four cases shown in Fig. 3e; in the fourth tumor (crcA5YX), genome duplication (GD) occurred immediately before clonal expansion, and we could not further narrow down the time window of the *PTPRK*-*RSPO* fusion.

In a recent study of kidney cancer [21], timing of alterations was estimated in years and the occurrences of alterations could be mapped to patient age. This resulted from the fact that somatic SNVs in kidney cancer are predominantly driven by aging-related mutational signatures (COSMIC signatures 1 and 5) and the mutation rate was shown to be constant over time. In addition, multiple tumors were sequenced for each patient in the kidney cancer study, so that mutation rates could be determined based on the phylogenetic trees of the tumors. In our cohort, the mutation rates are unlikely to be the same across patients and are certainly not constant over time, since some tumors are hyper-mutated and multiple mutational signatures (e.g., homologous recombination deficiency and COSMIC signatures 17 and 18, of unknown etiology) contribute to shape the mutational landscape. Therefore, the estimated timing in our study can only accommodate the relative order of genetic alterations and clonal expansions.

### Non-neutral evolution

**Fig. 4:**
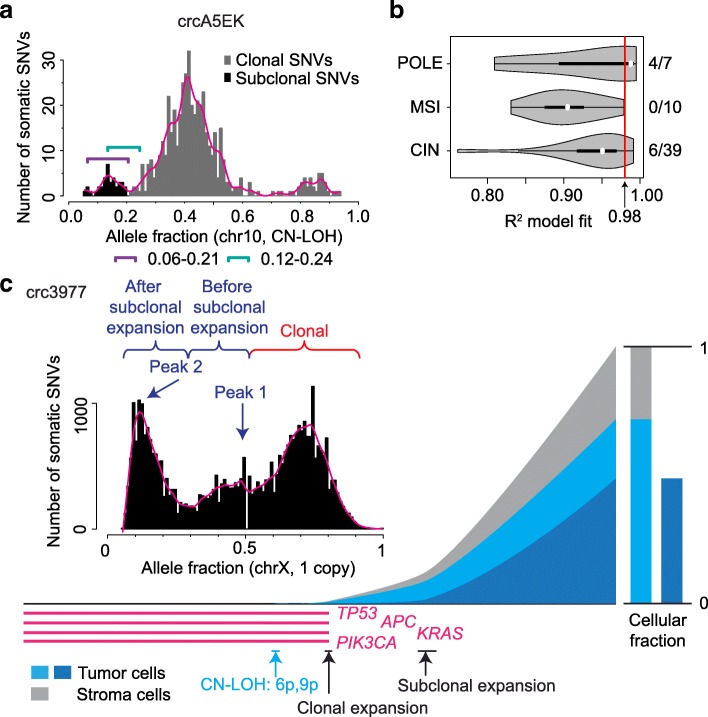
Non-neutral evolution. **a** Allele fraction distribution of chromosome 10 in tumor crcA5EK. SNVs in black are subclonal SNVs between an allele fraction range of 0.06 to 0.21. Allele fraction range of 0.12–0.24 used in Williams et al. is shown with a green bracket. **b** Violin plot of correlations between inverse allele fraction and cumulative number of SNVs. The red vertical line represents the 0.98 cutoff used in a previous study [14]. The numbers of tumors fitting the neutral evolution model and the total numbers of tumors tested are listed on the right for three subtypes. **c** An example of subclonal expansion. Two peaks of subclonal SNVs are present in the SNV allele fraction distribution plot. A subclonal expansion corresponding to peak 1 could be quantified since it is not too close to the SNV detection limit

The above analysis involved carefully distinguishing clonal and subclonal SNVs and accounting for sequencing depth, tumor purity, and ploidy in each sample. For example, we used somatic SNVs in genomic regions with either one or two copies so that clonal and subclonal SNVs were better separated, and then determined the upper bounds of allele fraction that best separated clonal and subclonal SNVs. The lower bounds were determined by testing SNV detection limits under different coverages (see the “Material and methods” section and Additional file 2: Figure S6a). For sample crcA5EK, for instance, the appropriate range of subclonal SNVs was 0.06–0.21, which appeared to optimally separate clonal and subclonal SNVs according to the distribution of allele fractions (Fig. 4a).

An important consequence of differentiating clonal and subclonal SNVs is that we are able to better understand the evolutionary process of tumor growth. A recent study proposed that a significant portion of colorectal cancers undergo neutral evolution after an initial clonal expansion [14]. Their theory was based on the simple argument that the allele fractions of subclonal SNVs should follow a power-law distribution if the tumor evolves neutrally. Given that mutations are introduced at every cell division, late-occurring SNVs in a neutrally expanding cell population should be present in more cells (as more cells acquire SNVs independently) but at lower allele fractions (a smaller number of cells carrying each SNV). Their model results in a simple relationship between the number of subclonal SNVs and the inverse of allele fractions, where a linear relationship corresponds to neutral evolution. Using an *R*^2^ value of 0.98 as a cutoff threshold for the linear relationship, they found that many colorectal cancers evolve neutrally. However, others have pointed out that many other evolutionary models besides neutral evolution are also compatible with their observed data [23].

In Williams et al., a single allele fraction range (0.12 to 0.24) was used to select subclonal SNVs for all tumors in the study, regardless of their sequencing depth, purity, and ploidy. In the example shown in Fig. 4a, it can be seen that variants in this range included many clonal SNVs and excluded many subclonal SNVs. Testing the tumor growth model for the new set of subclonal SNVs using the same mathematic formula from Williams et al., we found that this tumor no longer fit the neutral model (an *R*^2^ of 0.76 was below their threshold of 0.98). In addition, Williams et al. claimed that aneuploidy did not affect their model, but we observed the contrary. As pointed out recently [24], in aneuploid regions, clonal SNVs had much lower allele fractions (Additional file 2: Figure S6b), and so SNVs in the 0.12–0.24 range were mostly clonal. After selecting the appropriate subclonal SNVs on non-aneuploid chromosomes for all tumors and performing the same power-law distribution test, we found that only 6 out of 39 CIN tumors fit the neutral model (Fig. 4b, Additional file 1: Table S3), in sharp contrast to the previously reported number (31/82; *P* = 0.012, Fisher’s exact test). Furthermore, sometimes peak(s) of subclonal SNVs could be clearly observed (Fig. 4a), which is consistent with expansion of subclone(s). If subclonal SNV peaks were well separated and not too close to the detection limit, the timing of one of the subclonal expansions could also be quantified. Similar to the timing estimations of copy gain and clonal expansion demonstrated in Fig. 2b, we could further assign subclonal SNVs into two categories: before (SNVs around peak 1) and after (SNVs around peak 2) subclonal expansions. The timing of subclonal expansion was subsequently estimated based on the proportion of SNVs before and after subclonal expansion (Fig. 4c). In summary, our results demonstrated that, in a vast majority of the colorectal tumors, tumor growth is not neutral after the initial clonal expansion and additional selection on subclones is often present.

### Different mechanisms act on different genomic regions to optimize DNA dosage

**Fig. 5:**
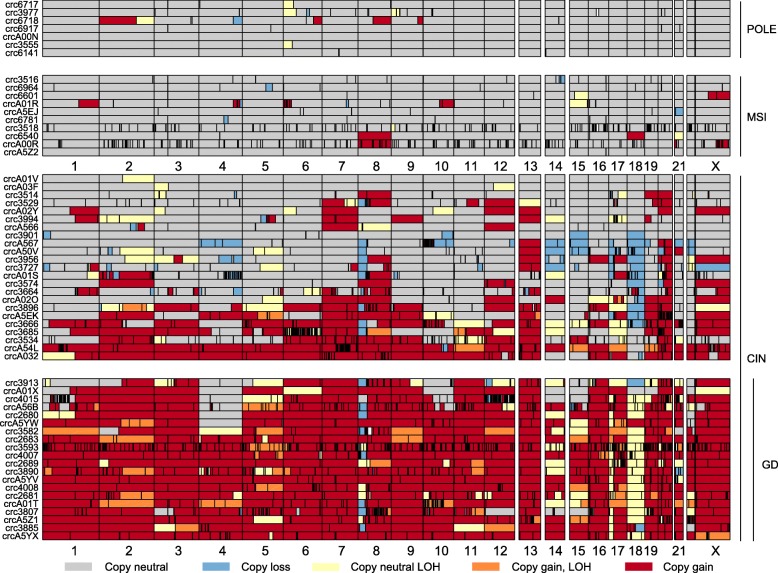
Copy number profiles of colorectal tumors. Each row represents one tumor. CIN tumors with GD are grouped together. “Copy gain, LOH” indicates three or more copies of the chromosomes are of the same parental origin

The integer copy numbers and the timing of their changes we inferred allowed us to better understand the process of copy gains and losses (Fig. 5). In a routine CNV analysis, identification of amplified and deleted regions is based on copy ratios between tumor and normal tissues, typically involving normalization of the copy ratios within each sample. This normalization becomes problematic when there are genome duplications (GDs), a common phenomenon in solid tumors [25]. For example, if most of the chromosomes in a tumor have three copies and only one chromosome has two copies, the two-copy chromosome would be considered a “loss” by a standard copy ratio approach because of its low dosage relative to other chromosomes. Furthermore, since GD is frequently followed by additional copy losses, it is not simple to distinguish such cases from the ones in which there are a series of duplications of individual chromosomes without GD. A previous approach for identifying GD was based only on allelic copy number imbalance [17], without considering the synchrony among the chromosomes.

We considered the fact that a full GD event gives rise to bi-allelic copy gains of all chromosomes at the same time (Additional file 2: Figure S7a), with copy loss prior to and after the GD manifesting as CN-LOH or a single copy gain after GD, respectively. To avoid calling sequential duplications as a GD, we required more than half of the genome to be duplicated (CN-LOH, one copy gain, bi-allelic two or more copy gain) at the same time since the number of chromosomes co-amplified seemed to follow a continuous distribution (Additional file 2: Figure S7b). In our cohort, 43% (20 out of 46) of the CIN tumors had GD events, while none of the *POLE*-mutant or MSI tumors exhibited GD, as expected (Fig. 5). In some GD cases, GD occurred quite early compared to clonal expansion (Figs. 2e and 3c, d). Among the non-GD CIN tumors, some had a substantial portion of their genomes amplified, but the duplications occurred at multiple time points (Additional file 2: Figure S7c).

We observed three distinct mechanisms acting on different genomic regions to optimize DNA dosage, as illustrated by three examples. First, we found frequent losses and CN-LOH events for chromosome 17p and 18 (where the major tumor suppressors, *TP53*, *SMAD4*, and *DCC*, reside) in CIN tumors (Fig. 5). Some tumors had additional copy gains after LOH of 17p, resulting in three or more copies of the same parental origin (orange color in Fig. 5). Chromosome 17p loss and CN-LOH were the most frequent arm-level copy changes across all cancers [26]. In our cohort, the loss of 17p only occurred in non-GD tumors; GD tumors were distinct, mostly having CN-LOHs but no copy loss (Fig. 5; *P* = 0.005, Fisher’s exact test). The occurrences of copy loss and CN-LOH on chromosome 18 were significantly correlated with GD status (Fig. 5; *P* = 1e−6, Fisher’s exact test): losses were only found in non-GD tumor with one exception, whereas all CN-LOHs were seen only in GD tumors. The whole chromosomal CN-LOH has been proposed to arise from chromosomal segregation errors in mitosis, whereas segmental CN-LOH can result from recombination during mitosis or DNA double-strand break repair [27]. However, the association between CN-LOH and GD in our data suggests that most CN-LOH takes place through a two-step process: a deletion followed by a duplication (often a GD).

Second, multiple losses may occur in GD tumors both before and after GD, as exemplified by chromosome 8. For that chromosome, most tumors only had one copy of 8p regardless of whether GD took place or not (Fig. 5, blue). The additional 8p loss must have occurred after GD because there was always one copy of 8p that remained and homozygous loss of 8p was never observed. Such a pattern suggested that there was an additional 8p loss after GD that provided further selective advantages to tumor cells, such as altering tumor metabolism [28].

Lastly, some chromosomes often had low dosage even after GD, not because of subsequent deletion but because they were not involved in GD and thus not amplified in the first place. For example, a previous study identified frequent loss of chromosomes 1p and 4 [2]. However, our analysis showed that they are amplified less frequently than being deleted after GD (Fig. 5). Of the 20 GD tumors, seven had two copies of chromosome 4, and six of them retained both parental copies. If these tumors had lost two copies after GD, we would have expected equal numbers of copy neutral and CN-LOH cases. However, the ratio of 6 to 1 suggested that a more likely scenario was that chromosome 4 was not duplicated during GD (*P* = 0.06 by binomial test is marginally significant due to the small sample size), although we cannot rule out the possibility of frequent loss of two copies of the same parental origin after GD. Furthermore, in non-GD CIN tumors, chromosome 4 is one of the rarely amplified chromosomes.

### Multiple independent occurrences of chromothripsis and kataegis

Two recently discovered mutational phenotypes are chromothripsis, a multitude of genomic rearrangements occurring on one or a few chromosomes as a result of a single catastrophic event [29], and kataegis, clustered occurrences of C > G and/or C > T SNVs associated with activities of APOBEC enzymes and genomic rearrangements [30]. Here, we define chromothripsis broadly as complex rearrangements that are likely to arise as a single catastrophic event. Chromothripsis was reported in a colorectal tumor in a cohort of four patients [31]; however, kataegis was not described in colorectal cancer yet. The TCGA colorectal cancer paper [2] analyzed 97 genomes sequenced at very low coverage (2–3×)—too low a coverage to detect chromothripsis and kataegis. In our cohort, we identified chromothripsis in 23 chromosomes from 13 tumors (Fig. 1, Additional file 1: Table S4, with more details in Additional file 2: Figure S8a) for all cases except the two described below. Of all the chromothripsis events, 20 were clonal and 3 were subclonal (Additional file 1: Table S4). Most of these tumors (12 out of 13) were CIN. One of them (crcA01R), however, was an MSI case, even though MSI tumors are considered chromosomally stable [2]. Although CNVs were rare in MSI tumors (Fig. 5) in general, their SV frequencies were comparable to those in CIN tumors (*P* = 0.82, Wilcoxon rank sum test). We suspect that the ancestor cells of MSI tumors may have less chromosomal mis-segregation (thus no aneuploidy) but may have similar levels of erroneous DNA replication and error-prone DNA double-strand break repair as CIN tumors.

**Fig. 6:**
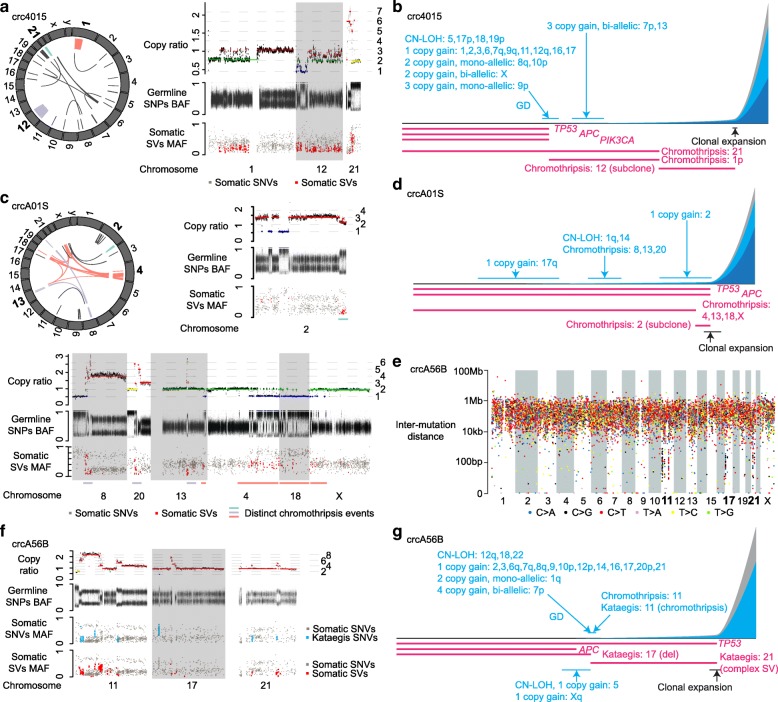
Timing of chromothripsis and kataegis. **a** Chromothripsis in crc4015. In the Circos plot on the left, the chromosomes with bold texts are predicted to have chromothripsis and the rearrangements belonging to different chromothripsis events are shown in distinct colors. Rearrangements not part of any chromothripsis are shown in black. The genetic alteration profiles on the right show chromosomes involved in chromothripsis. **b** Tumor progression map of crc4015. **c** Chromothripsis in crcA01S. In genetic alteration profiles on the upper right and at the bottom, regions involved in distinct chromothripsis events are marked by colored lines above the chromosome names. Two different regions of chromosome 13 are involved in two separate chromothripsis events. **d** Tumor progression map of crcA01S. **e** Rainfall plot for tumor crcA56B. The chromosomes with bold texts are predicted to carry kataegis. **f** Kataegis associated with chromothripsis, simple SV and complex SV. **g** Tumor progression map of crcA56B

Surprisingly, we found that chromothripsis could occur multiple times independently in a tumor. This was inferred based on the timing of CNVs and clonality associated with the chromothripsis events. An example is tumor crc4015, in which chromothripsis events were present on chromosomes 1, 12, and 21 (Fig. 6a, colored). First, the rearrangements were almost entirely intra-chromosomal, suggesting that the three chromosomes are unlikely to be related. Second, they displayed different mechanistic signatures and timing. For chromosome 1, our estimation of CNV timing showed that the oscillatory one-copy gain on 1p and the arm-level one-copy gain on 1q occurred at the same time as GD, consistent with a one-copy gain on the entire chromosome 1 followed by chromothripsis only on 1p. This chromothripsis perfectly recapitulated a micronuclei-induced event [32]. For chromosome 21, although its timing could not be estimated reliably due to the small number of somatic SNVs in the copy gain region, the chromothripsis event showed a different pattern: there were at least four copy states, some fragments were highly amplified, and a sub-telomeric region was lost. Such pattern is reminiscent of BFB-cycle-induced chromothripsis [33]. Finally, on chromosome 12, the event was subclonal (copy states are between integers) and appeared to have occurred later than the other two (Fig. 6b). Note that our chromothripsis mechanism inferences were based on the pattern of SV breakpoints and copy number profiles. We could not formally rule out other possible chromothripsis-forming mechanisms.

In another tumor (crcA01S), a detailed investigation of the chromothripsis events on chromosomes 2, 4, and 13 revealed that these were three independent events (Fig. 6c). The one at a sub-telomeric region of 2q was a subclonal event (Fig. 6c, right panel), with most segments having a copy number close to two but possessing a germline SNP BAF profile different from copy neutral or CN-LOH regions. More interestingly, there were two events that each involved multiple chromosomes. In one case, chromosomes 4, 13, 18, and X were part of a micronuclei-induced-chromothripsis with two copy states (Fig. 6c bottom). In the second case, chromosomes 8, 20, and another part of 13 were part of a BFB-cycle-induced chromothripsis event, displaying multiple copy states, high amplification of certain regions, and loss of sub-telomeric regions of chromosomes 8 and 20. This event occurred relatively early (Fig. 6d), as estimated via the two-copy gain of 8q, CN-LOH of 20p, and one-copy gain of 20q. Thus, our analysis suggested the presence of two clonal and one subclonal chromothripsis events, all having occurred independently, and two events involving different parts of the same chromosome (chromosome 13).

Kataegis events were present on 12 chromosomes across eight CIN tumors (Fig. 1 and Additional file 1: Table S5). In addition to the canonical kataegis SNVs (C > G and C > T), we also found C > A SNVs to be abundant (Additional file 2: Figure S8b crc4015). Determining the timing of kataegis based on kataegis SNV MAFs and the timing of associated somatic SVs (as kataegis SNVs are generated during SV formation), we found that kataegis sometimes occurred multiple times independently in a single tumor, as chromothripsis did. In one tumor (crcA56B; Fig. 6e), kataegis was found on chromosomes 11, 17, and 21 and was associated with chromothripsis, a simple deletion, and a complex rearrangement, respectively (Fig. 6f, blue). On chromosome 11, the copy numbers of segments mostly differed by ~ 1, suggesting that chromothripsis and its corresponding kataegis occurred after a GD. On chromosome 17, we did not detect somatic SVs nearby, but the kataegis SNVs were in close proximity of a copy loss region of 17p. The two-copy difference between the deleted region and its surrounding regions as well as the high MAFs of kataegis SNVs on chromosome 17 suggested that this kataegis event arose before the duplication of chromosome 17. Similarly, the copy changes and low MAFs of kataegis SNVs on chromosome 21 suggested that this kataegis occurred after a one-copy gain. Thus, we inferred that three independent kataegis events occurred at different times in this sample (Fig. 6g). Other kataegis cases are shown in Additional file 2: Figure S8a and S8b.

These results demonstrate that, although chromothripsis and kataegis are considered as bursts of genetic alterations that arise from a single catastrophic event, they can occur independently multiple times during tumor progression, sometimes by distinct mechanisms.

### Enhanced genetic model for colorectal cancer

**Fig. 7:**
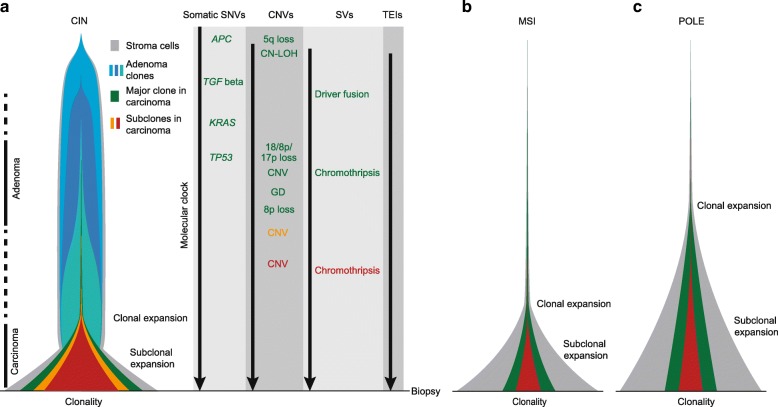
Tumor progression model. **a** CIN tumors. The representative timing of different types of genetic alterations is shown on the right. Tumor progresses from the top to the bottom, when the biopsy is taken. Genetic alterations that belong to different clones are shown in corresponding colors. **b** MSI tumors. **c** *POLE*-mutant tumors

With our in-depth and unbiased bioinformatic analysis of 63 WGS cases, we estimated timing of various genomic alterations in the history of colorectal tumors and detailed the many complex patterns previously unexamined. The frequently observed events were summarized into an enhanced genetic model in Fig. 7. For CIN tumors, *APC* mutation and 5q loss were the earliest driver alterations (light blue in Fig. 7a). CN-LOH can occur quite early, but the 5q CN-LOH always occurred after *APC* mutation so that both copies of *APC* were mutated. As an early-stage adenoma turned into a late-stage adenoma, *TGF* beta, *RAS*, and *TP53* were mutated sequentially, and 8p, 17p, and 18 were lost over time (dark blue and light green clones). Driver fusions, such as those involving *TCF7L2* or *RSPO*, also occurred in this time window. Eventually, one of the adenoma cells (dark green) took over the entire tumor cell population, out-competing other cells. After *TP53* mutation and loss of 17p, chromosome-level CNVs started to accumulate, sometimes with the emergence of GD. In GD tumors, 8p was frequently lost after the GD. The major clonal expansion happened fairly late, often preceded by subclonal copy changes. When the major clonal expansion occurred, adenoma started to transition into carcinoma. Tumor growth was usually not neutral after the major clonal expansion and secondary subclonal expansions were often present. Chromothripsis took place multiple times in the same cells, sometimes in subclones, but always after *TP53* inactivation. Some somatic TE insertions could also occur quite early, given their high allele fractions. Note that all tumors in our cohort are carcinomas and the order of genetic alterations in adenoma phase is adopted from the classical genetic model [8].

The timing of most genomic alterations could not be precisely estimated in MSI and *POLE*-mutant tumors since they had few copy changes (Fig. 7b, c). The clonalities of MSI and *POLE*-mutant tumors were lower than CIN tumors, and the major clonal expansion in *POLE*-mutant tumors started earlier than in MSI and CIN tumors.

Four different tumor evolution models have been proposed based on numerous studies in the past several decades: linear, branching, neutral, and punctuated (reviewed in Davis et al. [34]). The linear model posits that driver mutations are acquired slowly one by one and selective sweeps occur to form dominant clones; the branching model states that multiple clones emerge in parallel and diverge; the neutral model proposes that there is no additional selection after tumor initiation and that tumors evolve neutrally; and the punctuated model favors a large number of mutations burst in a short time. Our results reveal that colorectal tumors evolve as a mixture of at least three models. For CIN tumors at early stage (Fig. 7a), the *APC*, *KRAS*, and *TP53* mutations and arm-level copy losses occur one after another, and the dominant clones carry all of these driver alterations—so the linear model is in action at early stage. Then, parallel branches (subclones) emerge before the major clonal expansion and persist through late stage—therefore, the branching model is operating later. Bursts of mutations—in accordance with a punctuated model—arise in some tumors in the form of chromothripsis and kataegis. Therefore, these three evolutionary models could be operating at different stages in the same tumor, and one model is unlikely to fit all. In summary, our enhanced genetic model for colorectal cancer is substantially more comprehensive than the classical genetic model and offers further insights into the landmark events as well as their complexity in colorectal tumor progression.

## Discussion

Our ability to differentiate subclonal copy changes largely depends on the quality of copy number calls and the size of subclonal copy changes, as regions with copy ratios between two integer copy numbers are considered to have subclonal copy changes. Therefore, the fact that the colorectal genomes were profiled using WGS and copy number changes were identified at high resolution was critical. The noise level of copy number profiles also depends on sample quality and sequencing quality, and hence, fresh-frozen tissue and PCR-free sequencing libraries should be used whenever possible to reduce noise. For copy number changes, subclones could be still detected even when the coverage was not high if the data quality was high: for example, sample crc3885 was sequenced only at 31× coverage, but we could detect subclones easily because it had relatively little noise in its copy number profile (Additional file 2: Figure S5). If the copy number profile is noisier (e.g., sample crc3514 sequenced at 41×), we could still detect subclones but their copy changes had to involve multiple chromosomes (Additional file 2: Figure S5). The detection of subclonal SNVs depends more sensitively on sequencing depth because enough reads are needed at a given genomic location to capture the variant with a low allele fraction. In our cohort, the median coverage was ~ 60× for tumor (Additional file 1: Table S2, S3), which means that at 50% purity, somatic variants in 20% of the cells would correspond to 3 reads. We suspect that, depending on the purity, the minimum allele fraction would be roughly in the 0.1–0.2 range. Nine of the 64 had low coverage (30–35×), but eight of them had high purity (> 0.7), thus making it possible to detect a substantial fraction of subclonal SNVs (Additional file 2: Figure S6a).

Regarding timing estimation using somatic SNV MAFs, the peaks of MAF will be overlapping and truncating when the tumor purity is low. In such cases, the peaks of amplified and unamplified SNVs in MAF profiles are usually well separated, so the timing estimation of copy gain is still feasible. The unamplified SNVs in major clone are sometimes indistinguishable from subclonal SNVs (e.g., Figure 2e, f). Therefore, we only use one-copy and two-copy regions to estimate timing of clonal expansions. We note that the timing of clonal expansion shall be interpreted with caution, because we do not have the power to resolve the exact topology of subclones with bulk sequencing data. However, in our cohort, subclones detected by CNVs suggest that most subclones have nested phylogeny rather than a branching phylogeny, because the sum of two subclones shall not be bigger than the major clone (pigeonhole principle). For example, in crc2683 (Fig. 2i), the green clone must be nested in the orange clone and the light blue clone must be nested in the green clone. Other tumors show similar patterns (Fig. 3a–e and Additional file 2: Figure S4). Comparing MAF profiles across different tumors, we observed some tumors having more subclonal SNVs than others (Additional file 2: Figure S3c), which suggests different time of clonal expansions. Comparing different regions within the same tumor, we could infer the relative orders of copy gains and clonal expansion regardless of the subclonal phylogeny. For example, in crc2683, two regions were characterized as CN-LOH with an extra copy gain (three copies of the same parental origin). The copy change on chromosome 1p was a clonal event while the one on chromosome 15 was a subclonal event (Fig. 2i) based on copy ratios. There were very few subclonal SNVs on chromosome 1p suggesting the clonal expansion was very late (Additional file 2: Figure S3d). The numbers of SNVs occurring before and after copy gains in both regions were very similar (Additional file 2: Figure S3d) suggesting the subclonal copy gain on chromosome 15 occurred shortly after the copy gain on chromosome 1p. Taken together, our results suggest subclonal copy changes can occur much earlier than the major clonal expansion. Our conclusion is consistent with the multi-region sequencing study of colorectal cancer [13]. Therefore, our timing estimation of clonal expansion is biologically meaningful.

## Conclusion

The classical genetic model of human colorectal cancer provides the orders of mutations in a few important genes (e.g., *APC*, *KRAS*, and *TP53*). The relative timing of other genetic alterations such as arm-level gains and losses as well as genomic rearrangements remains unclear. Here, we reconstruct the progression history of 63 primary colorectal tumors by detailed computational analysis of WGS data. Our enhanced genetic model of colorectal cancer significantly broadens our understanding of the tumorigenesis process.

## Material and methods

### Sample processing

Tumors were resected, flash-frozen, and shipped to a centralized processing center (Biospecimen Core Resource) for additional pathologic review and extraction of nucleic acids. See Additional file 1: Table S1 for details about the patient cohort.

### WGS

Illumina paired-end pre-capture libraries were generated according to the manufacturer’s protocol (Illumina Multiplexing_SamplePrep_Guide_1005361_D) with modifications as described in the BCM-HGSC Illumina Barcoded Paired-End Capture Library Preparation protocol. The complete protocol and oligonucleotide sequences are accessible at the HGSC website (https://hgsc.bcm.edu/sites/default/files/documents/Illumina_Barcoded_Paired-End_Capture_Library_Preparation.pdf). Genomic DNA (500 ng) was sheared into fragments that range in size from 300 to 800 base pairs with the Covaris S2 or E210 system (Covaris, Inc. Woburn, MA). Fragmented DNA was purified using a 0.75× volume AMPure XP (Beckman, Cat. No. A63882). Pre-capture Ligation Mediated-PCR (LM-PCR) was performed for 6–8 cycles using the Illumina IBC1-12 index primers and Phusion PCR Supermix HiFi (2X) (NEB, Cat. No. M0531L). Whole-genome libraries were sequenced on the Illumina HiSeq 2000 and 2500 platforms. Each library was loaded on three to six lanes of a flow cell to achieve target coverages of 30× for normal samples and 60× for tumor samples. MSI status of the adenocarcinomas was evaluated in a previous study [12].

### Sequence alignment, variant calling, and processing

Initial sequence analysis was performed using the HGSC Mercury analysis pipeline (https://www.hgsc.bcm.edu/content/mercury). The primary analysis software on the instrument produces .bcl files that were transferred off-instrument to the HGSC analysis infrastructure by the HiSeq Real-time Analysis module. Once the run was complete and all .bcl files were transferred, Mercury ran the vendor’s primary analysis software (CASAVA), which demultiplexed pooled samples and generated sequence reads and base-call confidence values (qualities). Sequence reads were aligned to the reference genome using BWA [35]. Picard and GATK [36] were used for duplicate marking and indel realigning.

Somatic SNVs were identified by MuTect [37] (v1.1.4), VarScan 2 [38] (v2.3.6) and MuSE [39] (v1.0). Variants from all three callers were merged, and caller-specific variants were removed. Indels were identified by VarScan 2, GATK and the Baylor pipeline [40]. Caller-specific indels were also removed. Copy number segmentations and copy ratios were calculated by BIC-seq [41]. Somatic SVs and TE insertions were predicted by Meerkat [42] and TEA [5]. Mutations and epigenetic silencing of *POLE* and the mismatch repair pathway were investigated previously [15].

### Ploidy, clonality, and timing

The sample purity and ploidy were initially predicted by Sequenza [18], based on the copy ratios between tumor and matched normal samples and BAFs of germline heterozygous SNPs. If there is no ploidy change in the tumor, all chromosomes have a copy ratio of 1 and a BAF of 0.5. When there is a ploidy change, the copy ratios and BAFs of the corresponding chromosomes change accordingly. For example, when tumor purity is 100%, a chromosome with a one-copy gain will have a copy ratio of 1.5 and a BAF of 0.33 (1/3 for minor allele) or 0.67 (2/3 for major allele). When a tumor is 50% pure, a one-copy gain chromosome will have a copy ratio of 1.25 and a BAF of 0.4 (2/5 for minor allele) or 0.6 (3/5 for major allele). Therefore, given the copy ratio and BAF profiles of all chromosomes, sample purity and ploidy can be estimated. However, multiple solutions often exist for the same observed data. For example, a sample with 80% of cells carrying a one-copy gain will have the exact same copy ratio and BAF profiles as a sample with 40% of cells carrying a two-copy gain.

To improve the purity/ploidy calls and to infer subclonal structures, we performed additional analysis using the MAFs of somatic SNVs. If a purity/ploidy call was correct, it should be supported by the distribution of MAFs. Suppose tumor fraction is denoted by *p* and normal cell fraction by 1 − *p*, and the numbers of chromosomes for major and minor alleles are denoted by *n*_1_ and *n*_2_. Then, the peak of the largest MAFs corresponding to somatic SNVs on amplified chromosomes would be positioned at *p* × *n*_1_/(*p* × (*n*_1_ + *n*_2_) + 2 × (1 − *p*)). For example, for a chromosome with a one-copy gain in a tumor tissue of 50% purity (*p* = 0.5, *n*_1_ = 2, and *n*_2_ = 1), we would see the peak of MAF at 0.4. Additional peaks would be observed if there is a complex aneuploidy history or if subclonal SNVs are present (see examples in the “Results” section). If the MAF distribution conflicted with purity/ploidy calls, we would adjust purity and ploidy. We then tested if the observed MAFs were consistent with the adjusted purity and ploidy calls. For example, if the prediction of 80% of the cells carrying a one-copy gain does not fit with the observed MAFs, we would test the possibility of 40% of the cells carrying a two-copy gain, 27% of the cells carrying a three-copy gain, etc. If no solution could be found, we would leave that sample or chromosome as unresolved. Sequenza does not provide subclonal predictions. For regions that could not be solved by giving an integer copy number in a major clone, we assigned the smallest possible number of copy changes and inferred clonality according to the copy ratio and BAFs. Similarly, the inferred clonality and copy number should also be supported by MAFs. If the samples (most of the hyper- and ultra-mutated samples) had no major copy changes, purity was inferred by somatic SNVs. There were three tumors for which we cannot reliably determine the clonality and ploidy (crc2691, crcA565, and crcA50T).

We then inferred the evolutionary history of each region using MAFs of somatic SNVs. For each given history and tumor purity, the peaks of MAF will have expected locations. For example, in crc3913, the tumor purity is 0.74. Then, the SNVs occurred before and after CN-LOH were expected to have MAF of 0.74 (two out of two copies) and 0.37 (one out of two copies). We compared the observed peak locations of MAF profiles with expected peak locations of the possible evolutionary histories and decided the most likely history. When the total copy number is small (i.e., ≤ 4), there are only a few possible evolutionary paths (copy loss, copy neutral, CN-LOH, one-copy gain, one-copy gain with LOH, mono-allelic two-copy gain, bi-allelic two-copy gain, or two-copy gain with LOH). It is relatively easy to decide one history vs. others. For example, chromosomes 2 and 9 of sample crc2683 were both characterized as four copies with LOH. Given the observed MAF profiles, we inferred that chromosome 2 had mono-allelic three-copy gain after losing one parental allele while chromosome 9 had CN-LOH and then a mono-allelic two-copy gain (Fig. 2h and Additional file 2: Figure S3a). Note that, sometimes, we could not find a history that fits with observed copy ratio and variant profile, such as chromosome 2 of crc3913. In such cases, complex history as well as subclonal copy changes are often involved. When the copy number is larger than 4, there will be numerous paths to achieve the observed copy number. We did not attempt to differentiate all possibilities, but only gave simple solutions, such as mono-allelic three-copy gain and bi-allelic four-copy gain.

The timing of copy gains and CN-LOH events were estimated based on somatic SNVs. Somatic SNVs that occurred before amplification have higher MAFs. A spline smoothing line was fitted to each allele fraction distribution profile. Peaks were assigned as clonal before or after copy number change and subclonal. The timing of amplification was calculated as the proportion of somatic SNVs before amplification, while the timing of clonal expansion was calculated as the proportion of clonal somatic SNVs out of total SNVs. Only regions of one copy or two copies are used to infer timing of clonal expansion so that the subclonal peaks in the somatic SNV MAF profiles are well separated from clonal peaks. A 95% confidence interval was given by 1000 boot straps. The occurrence intervals of driver SNVs and driver SVs were inferred based on MAFs (given as before or after amplification). See examples in the “Results” section. Our timing estimations were compared with the cancerTiming package [22] for CN-LOH, one-copy gain, and mono-allelic two-copy gain regions in CIN tumors.

In each tumor, after ploidy and timing of aneuploidy was determined, if more than half of the chromosomes (chromosomes 1 to 22 and X) could be attributed to CN-LOH, one-copy gain, bi-allelic two-copy gain, and other higher order of bi-allelic copy gains occurring at the same time, the tumor was determined to have a GD event. A chromosome arm was counted as a half chromosome. A similar approach was taken in a breast cancer study [20], in which duplication of as few as five chromosomes were defined as a GD. Here, we required a GD event to involve more than half of the genome.

### Test for neutral evolution

Somatic SNVs with allele fractions ranging from 0.4 to 0.6 in tumor crc6917 (79× sequencing coverage) were used for simulation in order to evaluate the SNV detection power at different allele fractions. The selected SNVs were assigned with allele fractions ranging from 0 to 0.2, uniformly. Reads in the BAM file spanning a specific SNV were randomly assigned with reference allele or alternative allele according to the allele fraction assigned to individual SNVs. Then, the resulting BAM file was down-sampled to 30×, 40×, 50×, 60×, and 70× coverages. The SNV calling pipeline was run on the simulated BAM file and all down-sampled BAM files. For each 0.01 allele fraction interval, the proportion of SNVs that could be detected was calculated.

For each tumor, regions with one or two copies were selected and were required to contain at least 500 somatic SNVs to model the allele fraction distribution. Tumors with no region satisfying the above criteria were left out from this analysis. The lower bound allele fraction was determined as the lowest allele fraction where 80% of the SNVs could be detected in the above simulation experiment. The upper bound was determined by modeling the valley of allele fractions in a histogram of SNV MAFs that best separate the clonal and subclonal SNVs. The SNVs with allele fractions within the lower and upper bounds were used to test for neutral evolution as previously described [14]. The same *R*^2^ cutoff of 0.98 was used to determine neutral evolution.

### Chromothripsis and kataegis

Chromothripsis events were predicted by ShatterSeek [43]. Briefly, in chromothripsis events, the deletion-like, tandem-duplication-like, and inversion-like rearrangements shall be random and interleaved. Permutation tests were performed to identify such rearrangement clusters followed by manual curation for all cases. We did not require copy number to oscillate between two or three states since a recent study showed breakage-fusion-bridge cycles can generate chromothripsis events with multiple copy states [33].

Kataegis chromosomes were identified as follows: Fisher’s exact test was performed for each chromosome by comparing the proportion of C->T/C->G SNVs that are close to each other (among top 3% by inter-mutation distance) and the proportion of these SNVs in the entire genome. A *P* value of 0.01 with Bonferroni correction was used to select the chromosomes with C->T/C->G clusters, and manual curation was performed for all cases.

### Quantification and statistical analysis

Software package R version 3.2.1 was used for all statistical analysis. Specific statistical tests were reported in the main text where *P* values were reported and all tests were two-sided. A *P* value of 0.05 was used as a significance cutoff unless otherwise stated.

## Additional files

Additional file 1: **Table S1.** Patients’ information. **Table S2.** Sequencing samples’ information. **Table S3.** Test for neutral evolution. **Table S4.** List of chromothripsis. **Table S5.** List of kataegis. (XLSX 27 kb)

Additional file 2: **Figure S1.** Amplification artifacts and *TCF7L2* fusion. **Figure S2.** CN-LOHs occurring early in several tumors. **Figure S3.** Timing estimation. **Figure S4.** Tumor progression maps of other tumors. **Figure S5.** Timing of subclonal copy changes. **Figure S6.** Subclonal SNV selection in tumor evolution modeling. **Figure S7.** GD and sequential chromosomal duplications. **Fig. S8.** Chromothripsis and kataegis. (DOCX 3297 kb)

Additional file 3: Review history. (DOCX 232 kb)

## Data Availability

All primary sequence files can be downloaded by registered users from the Genomic Data Commons (GDC, https://portal.gdc.cancer.gov/). Clinical data are available through the TCGA Data Portal. All coordinates are based on the hg19 human reference genome. Our software of timing estimation “Beehive” is available at https://zenodo.org/record/3358081 (DOI: 10.5281/zenodo.3358081) under GPL 3.0 license [44].
