# Supplementary material for: An enhanced genetic model of colorectal cancer progression history
Source: Genome Biol. 2019 Aug 15;20:168. doi: 10.1186/s13059-019-1782-4 (PMC6694562; doi:10.1186/s13059-019-1782-4)
Supplement: Supplementary file 2 — Figure S1. Amplification artifacts and TCF7L2 fusion. Figure S2. CN-LOHs occurring early in several tumors. Figure S3. Timing estimation. Figure S4. Tumor progression maps of other tumors. Figure S5. Timing of subclonal copy changes. Figure S6. Subclonal SNV selection in tumor evolution modeling. Figure S7. GD and sequential chromosomal duplications. Fig. S8. Chromothripsis and kataegis. (DOCX 3297 kb) [file 13059_2019_1782_MOESM2_ESM.docx]

**Additional Information**

for

**An enhanced genetic model of colorectal cancer progression history**

Lixing Yang, Su Wang, Jake June-Koo Lee, Semin Lee, Eunjung Lee, Eve Shinbrot, David A. Wheeler, Raju Kucherlapati, Peter J. Park


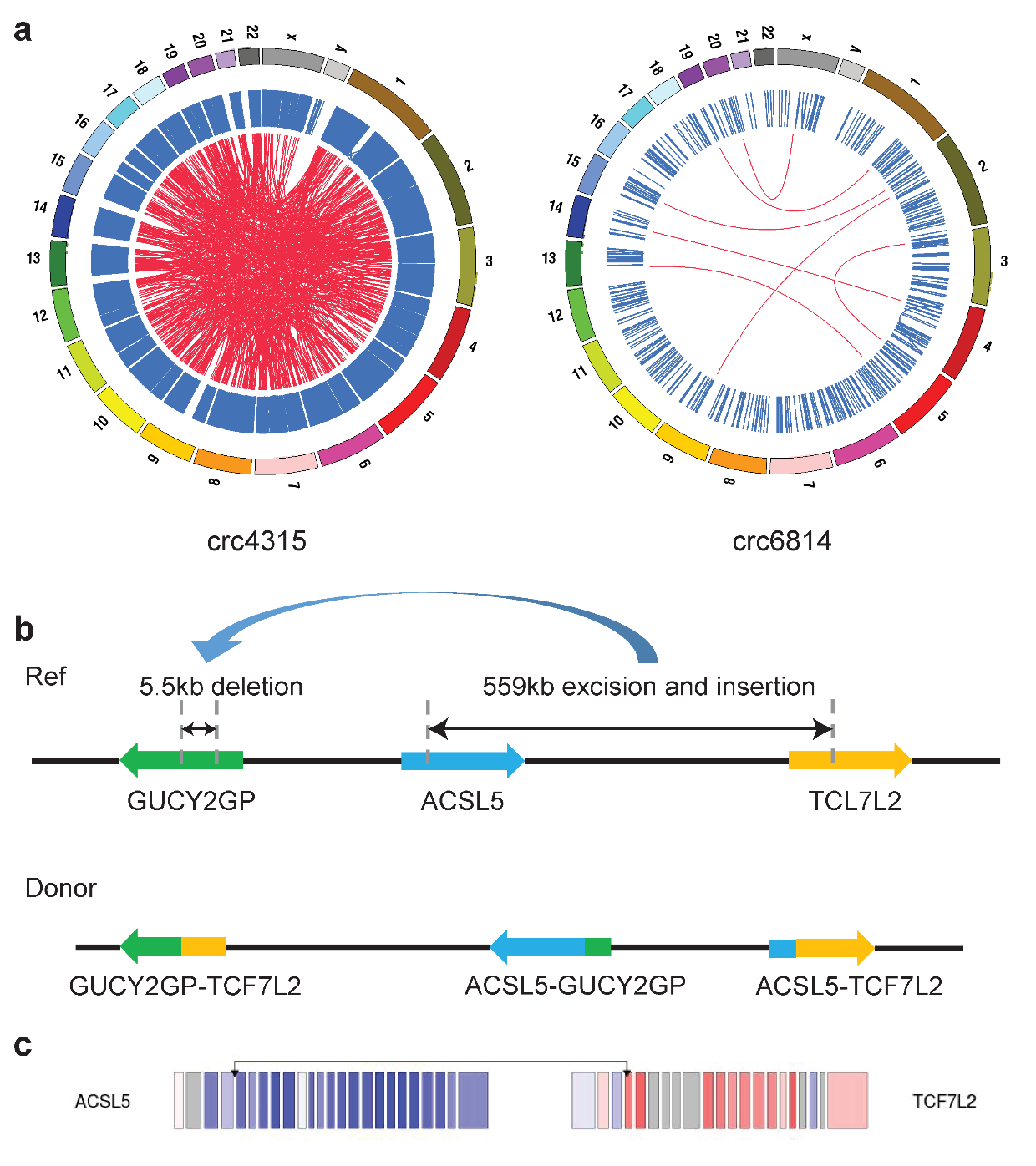


**Fig. S1. Amplification artifacts and *TCF7L2* fusion. a** Amplification artifacts in two tumors. Circos plots show somatic genome rearrangements in two tumors. Blue and red lines represent intra- and inter-chromosomal rearrangements. In these tumors, most rearrangements are small tandem duplications, and are likely to be amplification artifacts. These two tumors are discarded from further analysis. **b** Complex rearrangements resulting in in-frame *ACSL5*-*TCF7L2* fusion. A 5.5kb fragment of *GUCY2P* gene is deleted and a 559kb fragment spanning part of *ACSL5* and part of *TCF7L2* is flipped in orientation and inserted into the deletion junction. The complex rearrangements result in three gene fusions. **c** Heat map of exon specific expression of *ACSL5*-*TCF7L2* fusion. Colored boxes represent exons. Blue and red boxes indicate exons expressed at lower or higher expression levels relative to median expression in other tumors. Grey boxes indicate exons not expressed. Black arrows at top mark fusion breakpoints.


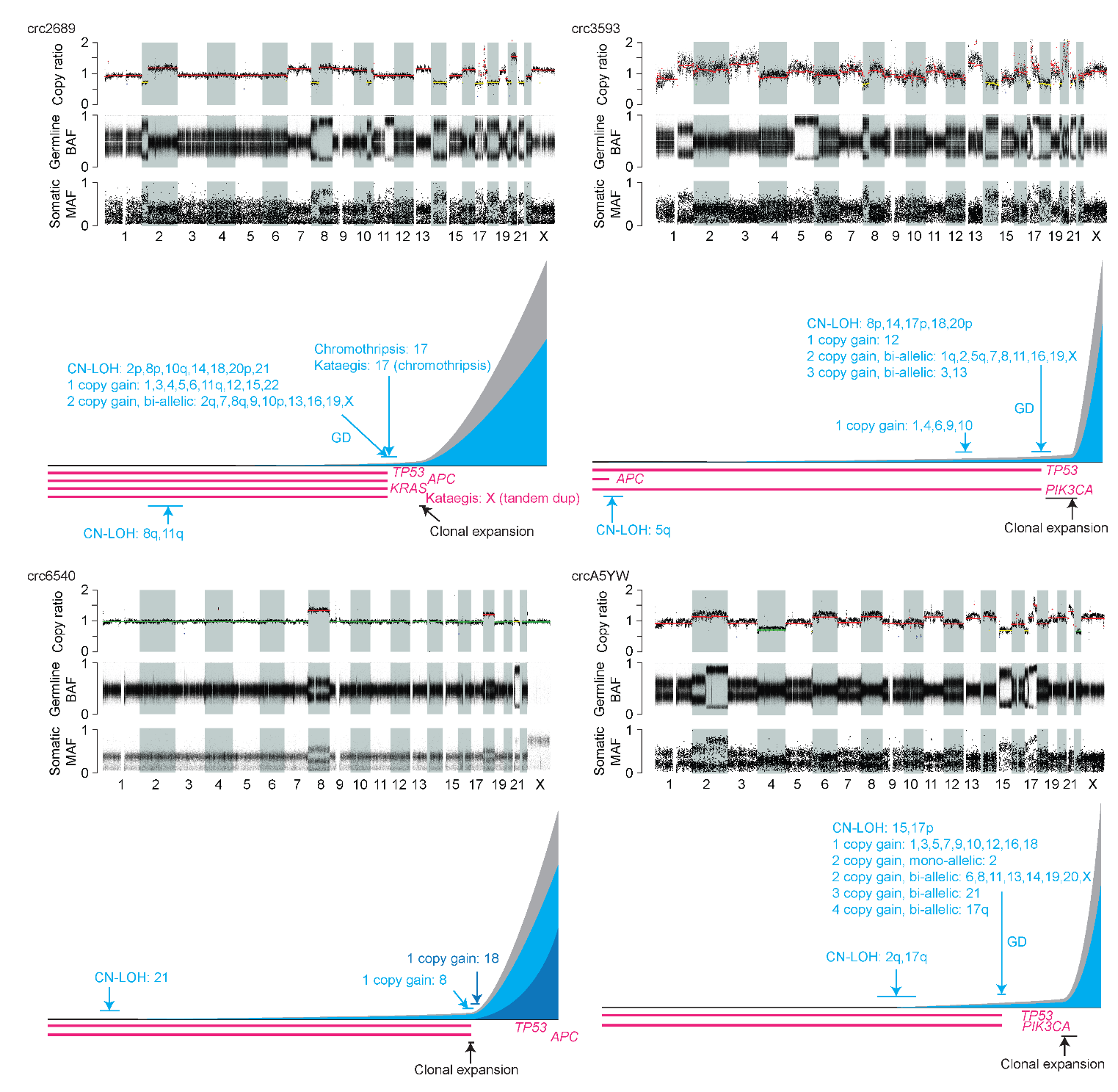


**Fig. S2. CN-LOHs occurring early in several tumors.** For four tumors, genetic alteration profiles are shown on the top and the tumor progression maps are shown at the bottom.


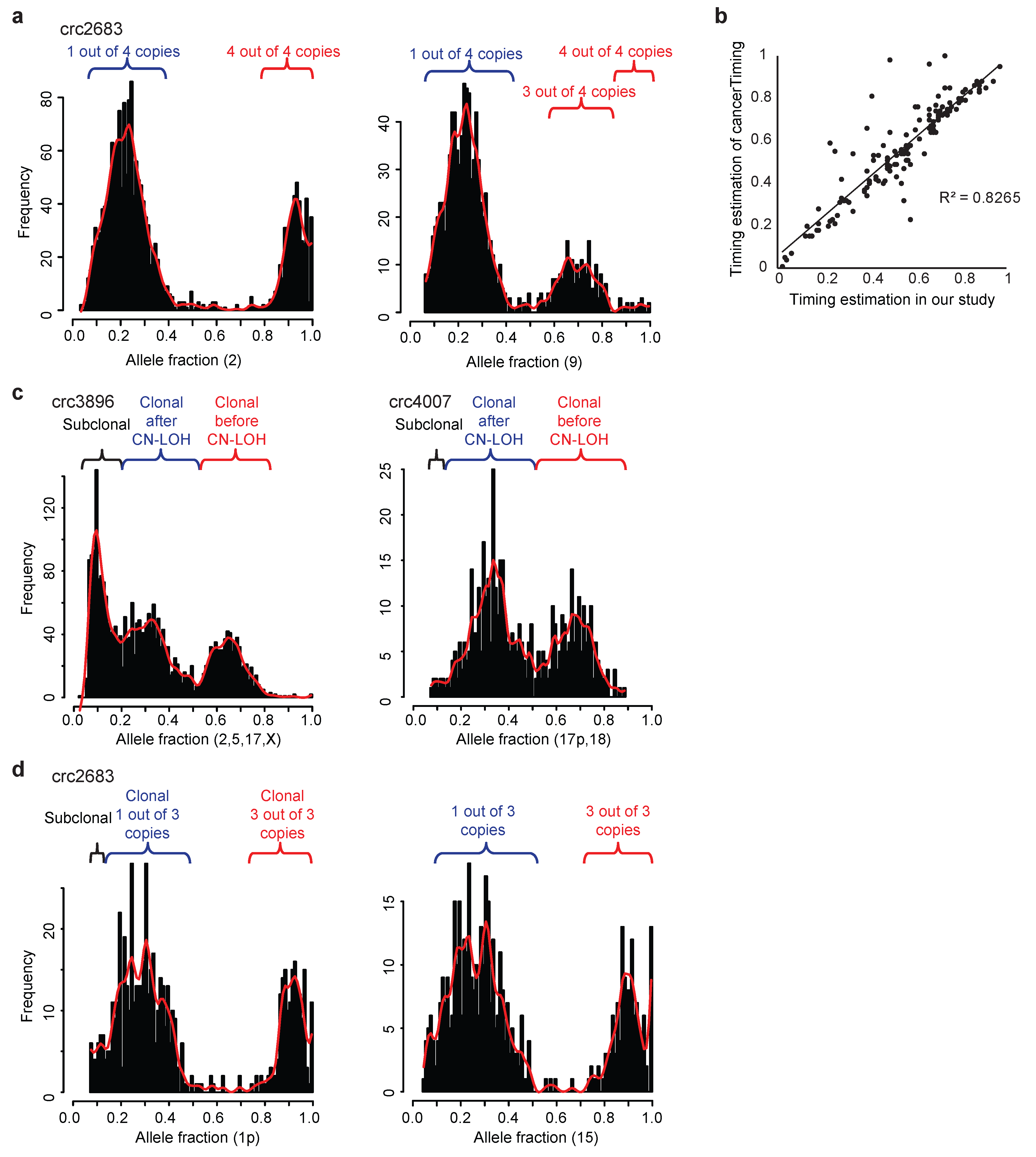


**Fig. S3. Timing estimation. a** MAF profiles for chromosomes 2 and 9 in crc2683. **b** Comparison of timing estimations in our study and using cancerTiming package. **c** MAF profiles for CN-LOH regions in crc3896 and crc4007. **d** MAF profiles for clonal and subclonal one-copy gain LOH regions in crc2683.


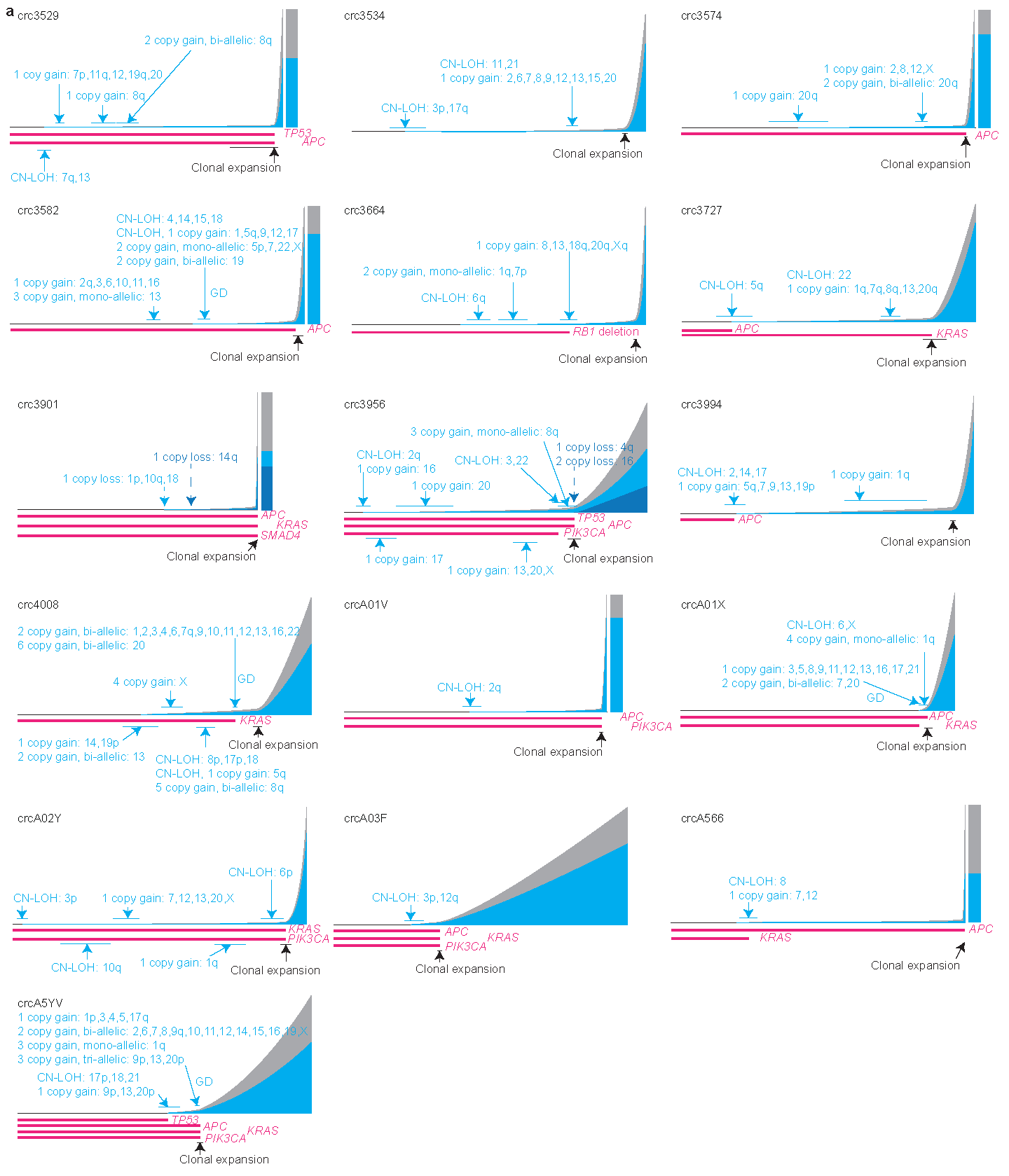


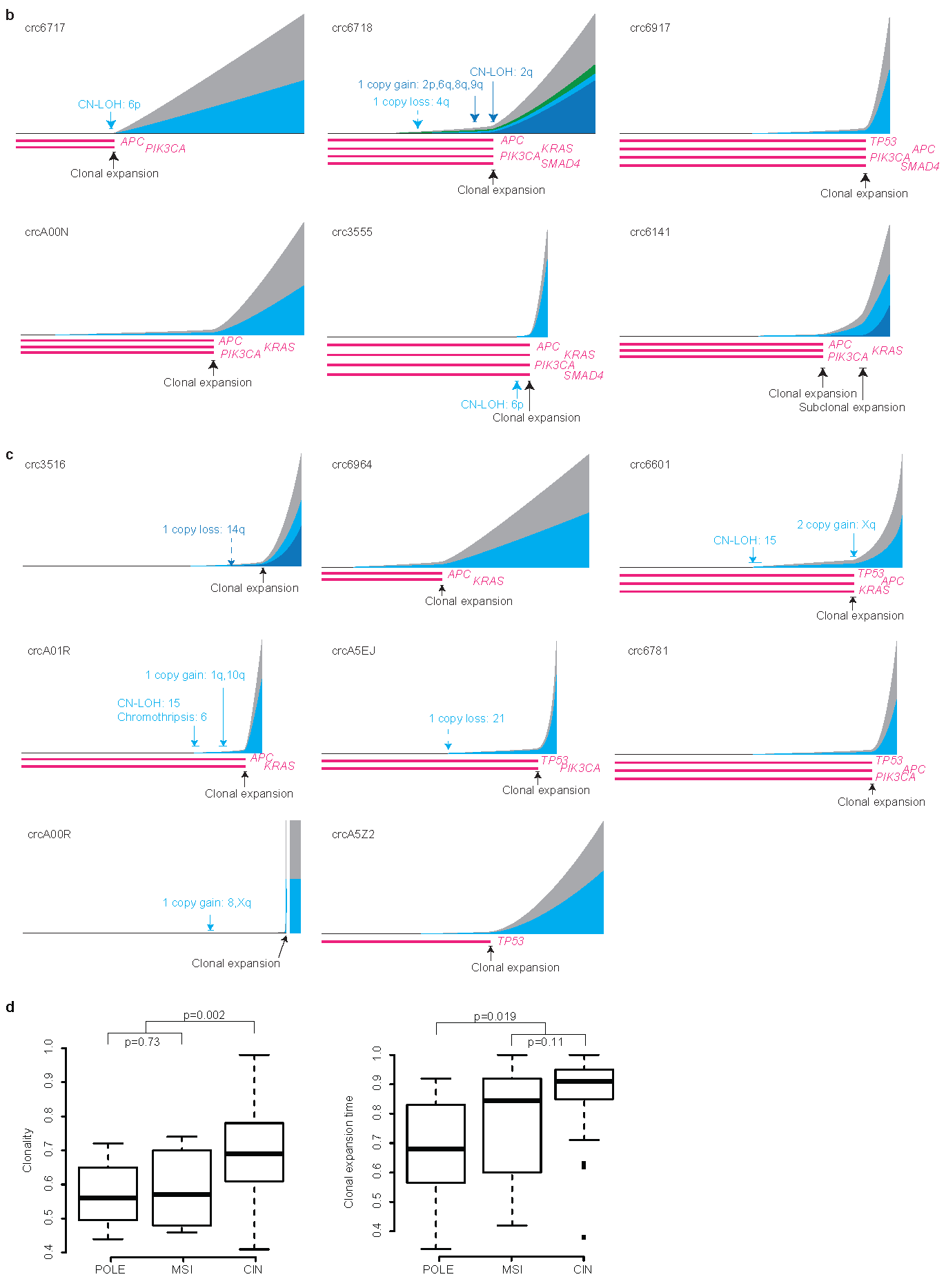


**Fig. S4. Tumor progression maps of other tumors. a** Tumor progression maps of CIN tumors. **b** Tumor progression maps of *POLE* mutant tumors. **c** Tumor progression maps of MSI tumors. **d** Comparisons of clonality and clonal expansion time for CIN, *POLE* mutant and MSI tumors.


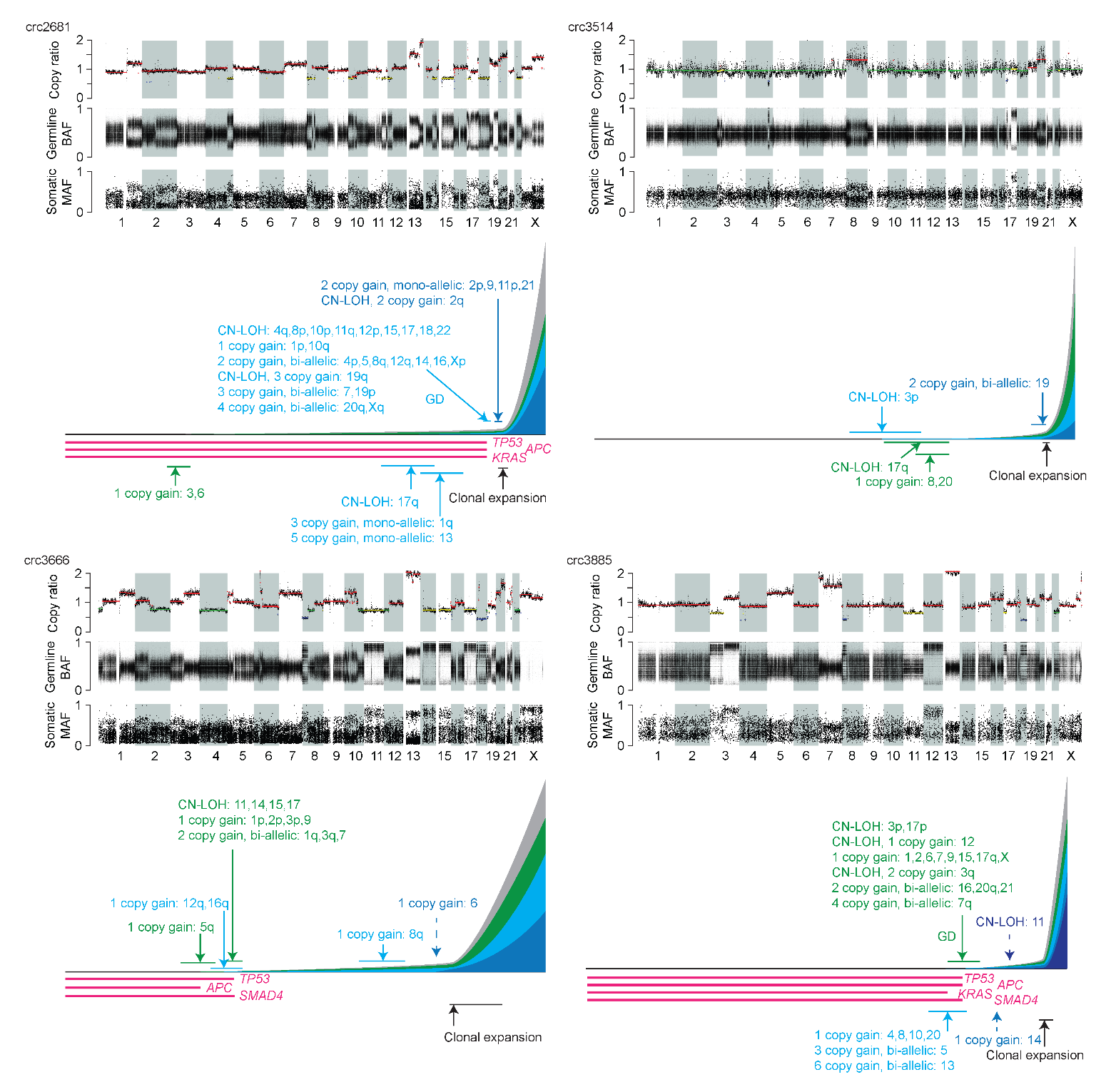


**Fig. S5. Timing of subclonal copy changes**. Genetic alteration profiles and tumor progression maps of four patients show subclonal copy changes occurring earlier than major clonal expansion.


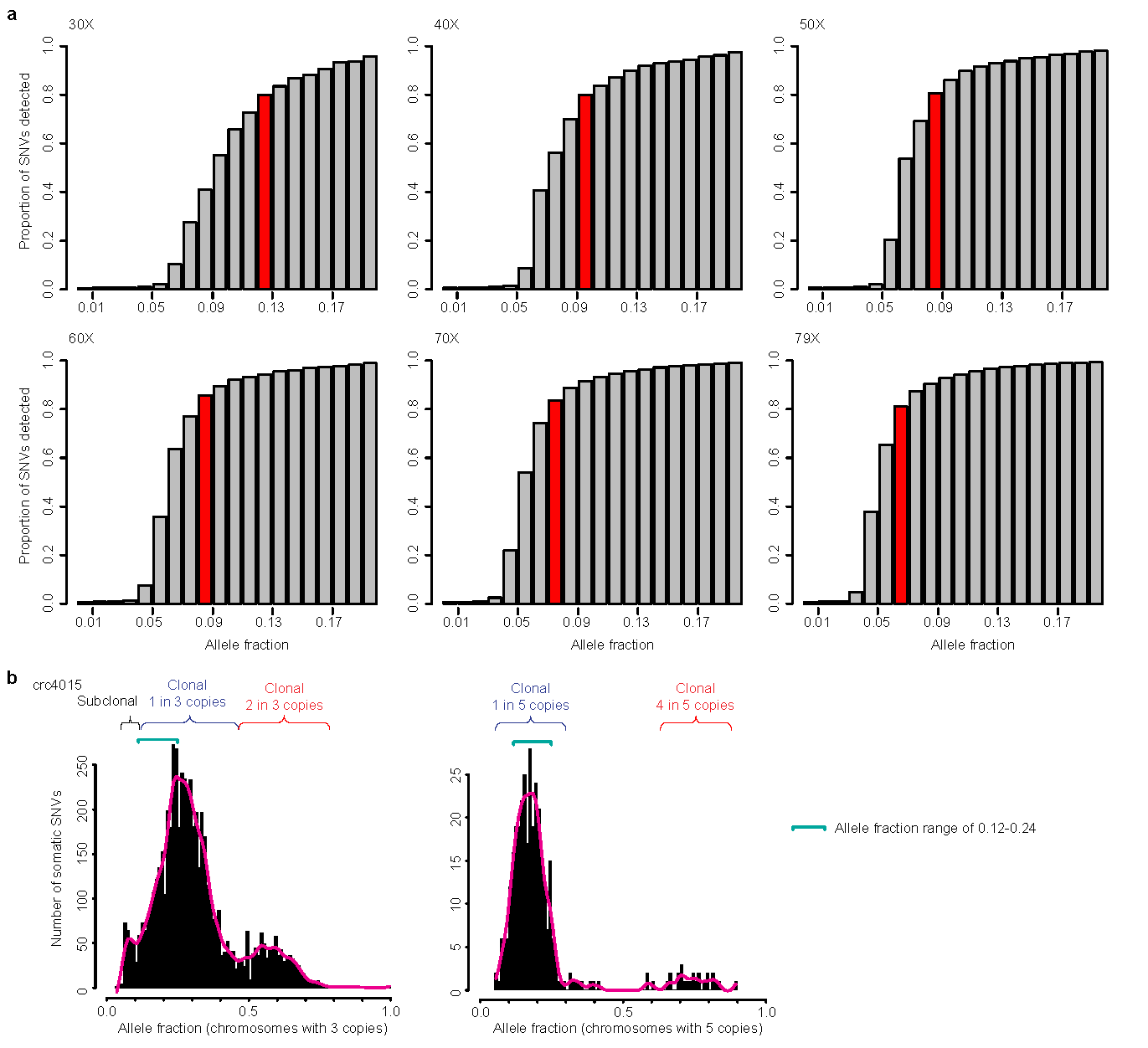


**Fig. S6. Subclonal SNV selection in tumor evolution modeling. a** Testing detection limit of somatic SNVs. Reads are down-sampled to test detection limit for somatic SNVs at different sequencing coverages. The red bars in each plot represent the lowest allele fraction with a minimum detection rate of 80%. **b** Subclonal SNV selection affected by ploidy. Allele fraction distributions for chromosomes with 3 copies and 5 copies in tumor crc4015. The SNVs in allele fraction range 0.12 to 0.24 (green brackets) are mostly clonal SNVs.


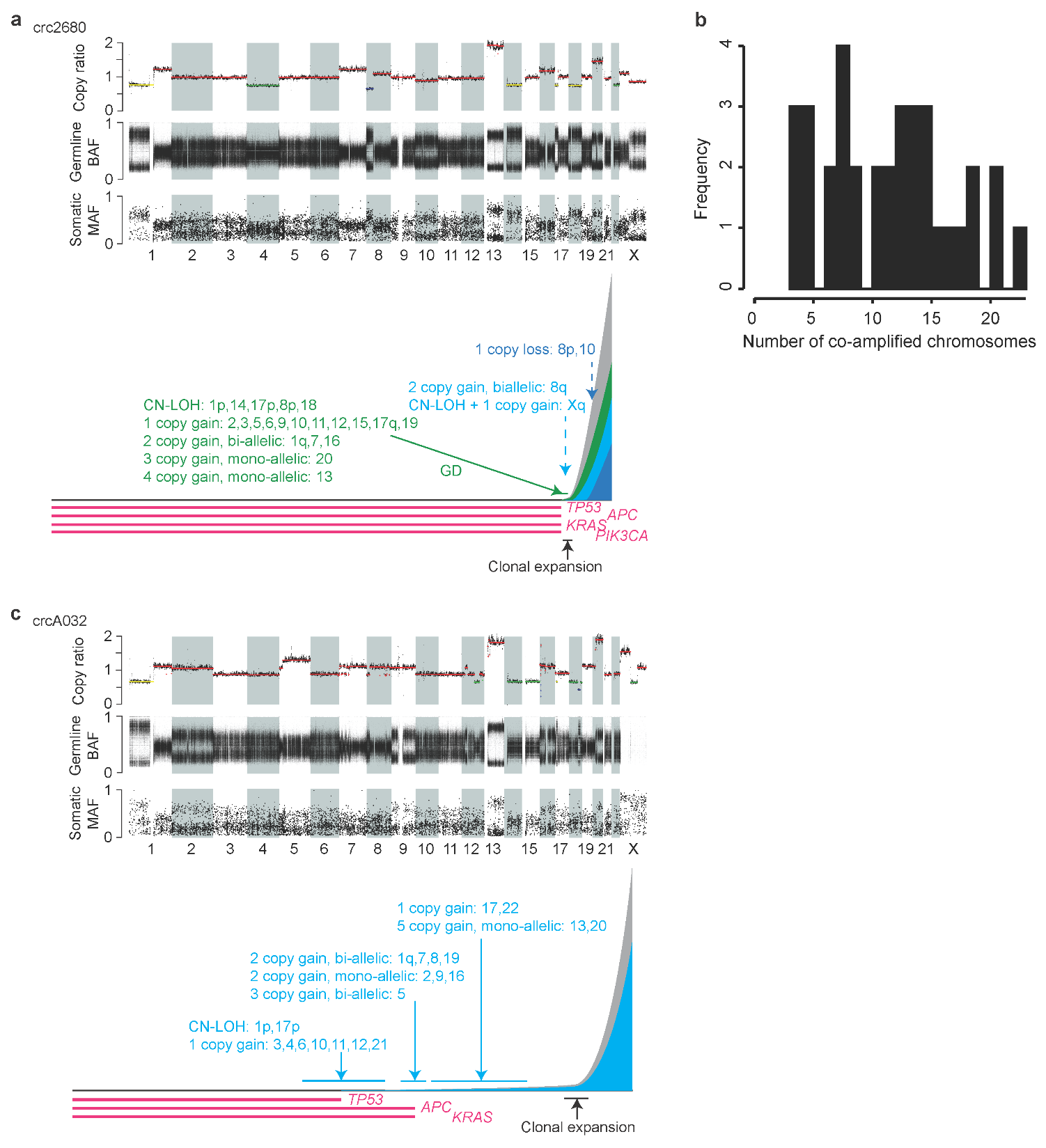


**Fig. S7. GD and sequential chromosomal duplications. a** GD in tumor crc2680. All clonal copy changes occurred at the same time. **b** Distribution of c-amplified chromosomes (>3 chromosomes) in CIN tumors. **c** Sequential chromosomal duplications at different time points in tumor crcA032.


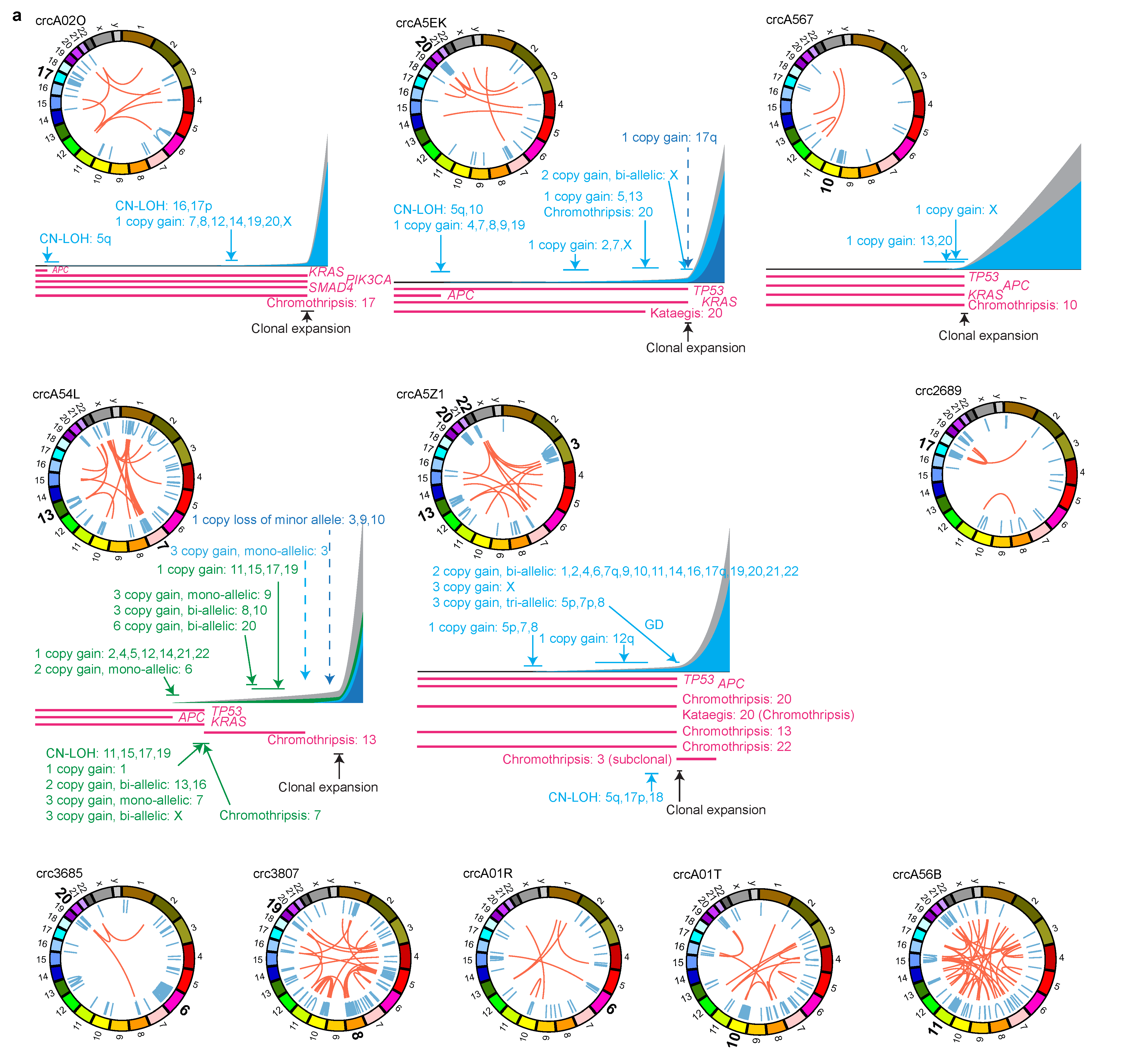


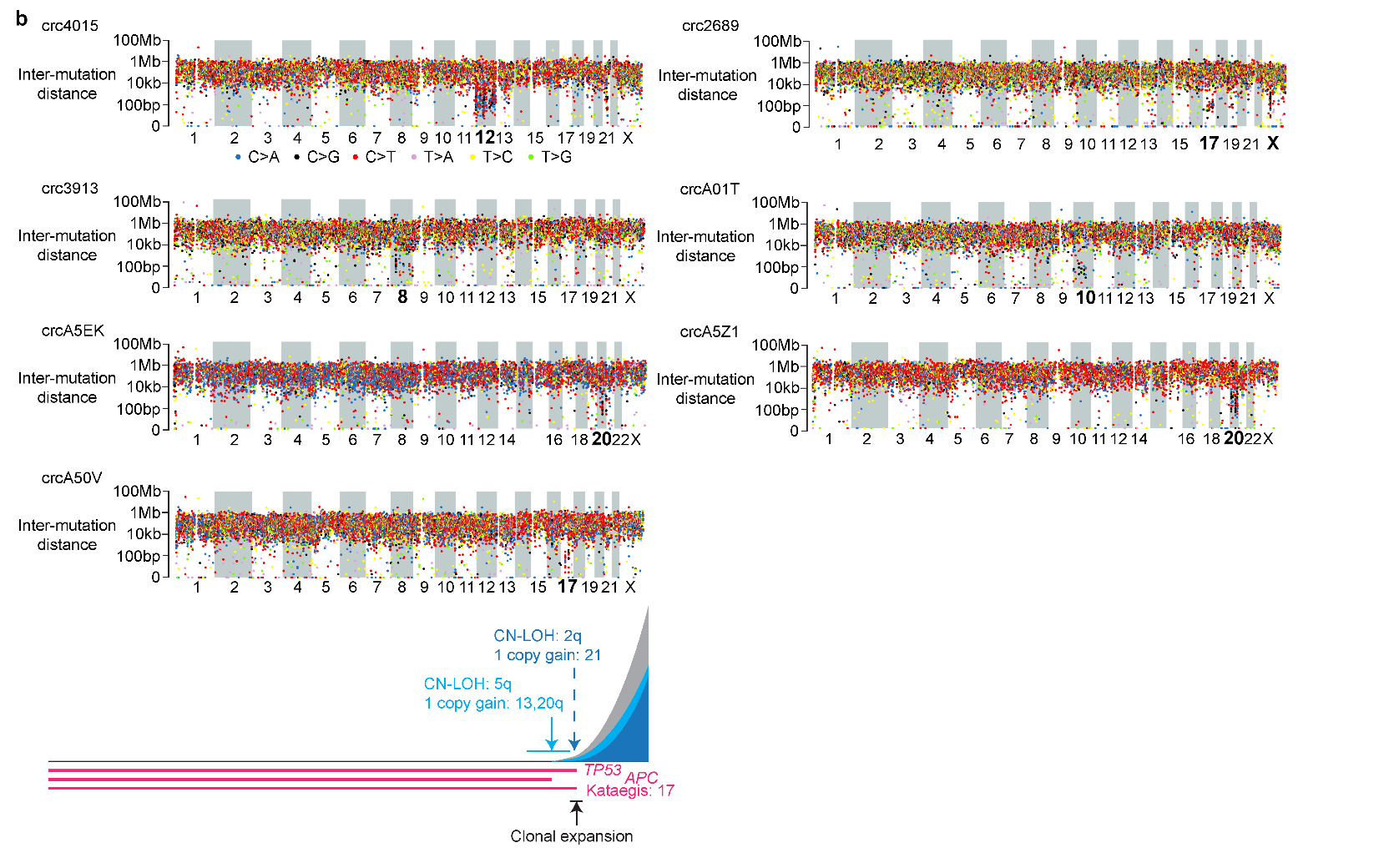


**Fig. S8. Chromothripsis and kataegis. a** Circos plots and progression maps of tumors carrying chromothripsis. Chromosome names in bold texts in Circos plots are the ones having chromothripsis. **b** Rainfall plots and progression map of tumors carrying kataegis. Chromosome names in bold texts in rainfall plots are the ones having kataegis.
