# Supplementary material for: An enhanced genetic model of colorectal cancer progression history
Source: Genome Biol. 2019 Aug 15;20:168. doi: 10.1186/s13059-019-1782-4 (PMC6694562; doi:10.1186/s13059-019-1782-4)
Supplement: Supplementary file 3 — Review history. (DOCX 232 kb) [file 13059_2019_1782_MOESM3_ESM.docx]

Review History

**First round of review**

**Comments to author and authors’ point-by-point responses to the reviewers’ comments:**

**Reviewer 1**

Yang et al present an analysis of the evolutionary history of 63 colorectal adenocarcinomas based on whole genome sequencing data. The authors use the allele frequencies of point mutations in relation to copy number alterations to time allelic gains and sub clonal variants. The analysis is comprehensive across a range of genomic alterations, including point mutations, chromosomal gains, chromothripsis and structural variants.
Such evolutionary timing, while not entirely novel, has not been applied widely in the past. Still the methodological novelty of the manuscript feels a bit exaggerated, especially since this rational has been applied in a pan-cancer analysis of more than 2700 genomes, (<http://dx.doi.org/10.1101/161562>). There is clear merit in this analysis though, as it discusses the evolutionary history of CRC in unprecedented detail.
However, a number of questions remains, especially since many of the analysis steps are either somewhat unsystematic or at least poorly described in the methods. I therefore am not fully convinced by many of the authors' claims and keep wondering whether alternative explanations such as different copy number solutions exist to explain the observed phenomena.
It could help trying to further emphasise specific trends of different alterations. In the given form the reader tends to be overwhelmed with a plethora of different examples and it is difficult to judge whether there are any clear evolutionary patterns.

*Response:* *We thank the reviewer for acknowledging the value of our work. We are well aware of the pan-cancer analysis of 2700 genomes, as we are also part of the Pan-Can Analysis of Whole-Genomes (PCAWG) effort. We believe that the two papers are complementary: whereas the pan-cancer work (still unpublished) summarizes the general features across each tumor type, we focused on a single tumor type in much greater depth. A pan-cancer-level summary is helpful for a big picture-view, but it misses many details and complexities. A general analytical framework for studying tumor evolution can only take us so far—we have found that careful curation of individual cases is essential, and this simply cannot be done for 2700 cases. In addition to copy number changes and point mutations, we have also estimated the timing of structural variants such as gene fusions and chromothripsis, which are clearly important for tumor evolution.*

*We do describe many different examples, and the methods may seem a bit unsystematic. However, we have found that there are different subtypes in our cohort and there is considerable heterogeneity within a subtype, necessitating that we adapt our approach for specific cases. To account for a wide range of alterations and illustrate their complexities, we do feel that going through many tumors is important. We have revised our manuscript to better clarify the methods.*

Major issues

Timing of clonal gains

It is a bit unclear how the author's method performs and their approach to modelling data appears a bit vague from a statistical point of view. There is no formal model of the distributions of variant allele frequencies (the authors fit splines instead, according to the methods). This is a bit surprising because the copy number profile implies defined frequencies for the expectations of point mutations. As a consequence, the process of assigning variants to different allelic copy numbers remains unclear and it becomes harder to decide whether a particular number of variants implies a particular evolutionary history or merely an error in the underlying copy number data. For example, is the solution proposed by the authors in Fig 2c for Chrome 9 really such that one of the two alleles after CN-LOH get two extra copies while the other none? Is that timing compatible with the GD (if GD is essentially a failed mitosis, then all alleles increase by 1). Could it be that instead the intermittent VAF band depicted resembles two instead of three copies, noting that the rescaled VAF can be somewhat skewed as evidenced by the early clonal band?
There is also no model to adjust for variant calling threshold, which can bias estimates, especially in low purity samples where the distributions are substantially overlapping and truncated.
Generally, the authors should demonstrate the accuracy of their approach either by simulations and/or by comparison to other tools. There are at least three alternative algorithms
PhylogicNDT (<https://doi.org/10.1101/508127>)
MutationTimeR (<http://dx.doi.org/10.1101/161562>)
CancerTiming (<http://dx.doi.org/10.1093/bioinformatics/btt546>)

*Response:**Let us first describe the specific example and then come back to the overall point. The reviewer was concerned about other possible histories of chr9 in Fig 2h (we believe the reviewer was referring to Fig 2h instead of 2c). To clarify this question, we now provide the histograms of MAFs for chr2 and 9 in Figure S3a (shown on the right; all supplement figures have been renumbered) in addition to the plot of the MAFs along the chromosome in the main figure (bottom left panel of 2h, “somatic MAFs”). Since the tumor purity is 0.98, the expected MAFs are 0.98 for 4 copies, 0.74 for 3 copies, 0.25 for 1 copy, if there was a mono-allelic 2 copy gain after CN-LOH. They would be 0.98 for 4 copies, 0.49 for 2 copies, and 0.25 for 1 copy if there was a bi-allelic 2 copy gain after CN-LOH. Based on the histogram of MAF (chromosome 9 is the right panel in the pasted figure), we are convinced that a mono-allelic 2 copy gain is more likely to be the case (a large bump around .74). We also think that it occurred during GD given all the other events that occurred in that tumor (Figure 2i). Indeed, based on our observation in many tumors, GD rarely generates bi-allelic copy gains for all chromosomes. That is why we used more than half of the genome duplicated as our criteria to define GD. We have discussed the possibilities of chromosome not duplicated and chromosome loss after GD in the manuscript. In this particular tumor crc2683, many chromosomes have bi-allelic copy gains during GD while many others have mono-allelic copy gains (Figure 2i).*

*We thank the reviewer for pointing out the three manuscripts/algorithms for comparison. The first two manuscripts are unpublished and we found some of the software packages hard to use. MutationTimeR (*[*https://github.com/gerstung-lab/MutationTimeR/blob/1f4754cde898ec76280c709cfc6214a329409964/MutationTime.R*](https://github.com/gerstung-lab/MutationTimeR/blob/1f4754cde898ec76280c709cfc6214a329409964/MutationTime.R)*) was used in the pan-cancer study for timing estimation, but we were not available to run it, as it does not provide instructions. PCAWG_timing.R (*[*https://github.com/clemencyjolly/PCAWG11-Timing_and_Signatures/blob/77619371c0cec71ec9e37f409d360bede7f95e33/PCAWG_timing.R*](https://github.com/clemencyjolly/PCAWG11-Timing_and_Signatures/blob/77619371c0cec71ec9e37f409d360bede7f95e33/PCAWG_timing.R)*), which as the name suggests is used in the pancancer manuscript, depends on package cancerTiming. Since cancerTiming is the only published algorithm, we performed detailed comparison between our method and cancerTiming.*

*In cancerTiming, sophisticated statistical modeling of variants is performed. But there are two separate issues regarding to the variant modeling: i) to infer the evolutionary history; and ii) to assign variants to a specific copy number and estimate timing. The evolutionary history in our study is reconstructed manually for each chromosome prior to timing estimation. cancerTiming algorithm does not infer evolutionary history either but requires user to provide as a matrix. As pointed out by the reviewer, specific history does define expected allele frequencies. We compared the observed peak locations with expected peak locations of the possible evolutionary histories and decided the most likely history. When the total copy number is small (e.g., <=4), there are only a few possible evolutionary paths (copy loss, copy neutral, CN-LOH, one-copy gain, one-copy gain with LOH, mono-allelic two-copy gain, bi-allelic two-copy gain or two-copy gain after LOH). It is relatively easy to decide one most likely history vs others. Note that, sometimes, we could not find a history that fits the observed copy ratio and variant profiles, such as chr2 of crc3913. In such cases, complex history as well as subclonal copy changes are often involved. When the copy number is larger than 4, there will be numerous paths to achieve the observed copy number. We did not attempt to differentiate all possibilities, but only gave simple solutions, such as mono-allelic three-copy gain and bi-allelic four-copy gain.*

*We did not employ sophisticated statistical modeling on assigning variants to specific copy state, because we found that statistical modeling has its limitations in dealing with more complicated cases. We have run cancerTiming in all of our CIN tumors with CN-LOH, 1 copy gain and mono-allelic 2 copy gain regions. Out timing estimations are almost the same as cancerTiming algorithm (R2=0.83). cancerTiming algorithm can only model a subset of evolutionary histories. Bi-allelic two-copy gain cannot be modeled since the matrix is not invertible. Some complex histories cannot be modeled either (e.g. 2 types of copy-gain LOH events shown in Fig 2h). Our spline smooth method can overcome these limitations and we think our method is superior to cancerTiming for our analysis. We also agree that when the tumor purity is low and peaks of the MAF distribution will be overlapping and truncating. However, in such cases, the peaks of amplified and unamplified SNVs in MAF profiles are usually well separated, so the timing estimation of copy gain is still feasible. The unamplified SNVs in major clone are sometimes indistinguishable from subclonal SNVs (e.g. Fig 2e and 2f). That is why we only use 2 copy or 1 copy regions to estimate timing of clonal expansions. We have revised texts in the Methods and Results sections to clarify our approaches, and added more texts in Discussion section to address the limitations.*

Timing of clonal expansion

It's not clear how this is performed and whether the inferred time points are accurate. How is the limited sensitivity modelled, which will lead to an underestimation of the latency and how is the subclonal phylogeny modelled (in linearly nested phylogeny the number of mutations per subclone would add, while in a branching topology the arithmetic would be sublinear). Also it remains unclear how subclonal copy number alterations where factored into the equation (these also come with their own expected allele frequencies of point mutations) and the author's proposed scenario of a long latency of subclonal copy number evolution followed by a sudden clonal expansion requires additional evidence and explanation.

*Response:* *The reviewer has made a very good point on the limitation of our clonal expansion estimation. We should have clarified more in our manuscript. As described in the previous section, we only used 2 copy or 1 copy regions to estimate the timing of clonal expansions because the subclonal SNV peaks are better separated from clonal SNV peaks. Only the regions without subclonal CNVs were selected. The timing estimation of clonal expansion is measuring the proportion of subclonal SNVs and should be interpreted with caution—we do acknowledge that we do not have the power to resolve the exact topology of subclones with bulk sequencing data, and we have added this point in the revised version.*

*Nonetheless, subclones detected by CNVs suggest that most subclones have nested phylogeny because the sum of two subclones should not be bigger than the major clone (pigeonhole principle). For example, in crc2683 (Fig 2i), the green clone must be nested in the orange clone and the light blue clone must be nested in the green clone. Other tumors show similar patterns (Fig 3a-3e and S4). Comparing MAF profiles across different tumors, we observed some tumors having more subclonal SNVs than others (Fig S3c), which suggests different time of clonal expansions. Comparing different regions within the same tumor, we could infer the relative orders of copy gains and clonal expansion regardless of the subclonal phylogeny. For example, in crc2683, two regions were characterized as CN-LOH with an extra copy gain (three copies of the same parental origin). The copy change on chromosome 1p was a clonal event while the one on chromosome 15 was a subclonal event (Fig 2i) based on copy ratios. There were very few subclonal SNVs on chromosome 1p suggesting the clonal expansion was very late (Fig S3d). The numbers of SNVs occurring before and after copy gains in both regions were very similar (Fig S3d) suggesting the subclonal copy gain on chromosome 15 occurred shortly after the copy gain on chromosome 1p. Taken together, our results suggest subclonal copy changes can occur much earlier than the major clonal expansion. Our conclusion is consistent with the multi-region sequencing study of colorectal cancer (Sottoriva et al. 2015). Therefore, we think our timing estimation of clonal expansion is biologically meaningful. We have added texts in Methods, Results and Discussion sections to address these concerns.*

Timing of kataegis

The timing of SVs based on their variant allele frequencies can be very challenging due to mapping issues. Why don't the authors use the variant allele frequencies of the point mutations, especially since only a fraction of clustered mutations occurs in the immediate vicinity of breakpoints? Do all mutations in a kataegis shower have compatible VAF?

*Response: We actually used the kataegis MAFs for inferences already. We pointed out that the point mutations of kataegis on chromosome 17 had high MAFs and the ones on chromosome 21 had low MAFs, which suggested that the kataegis on 17 occurred before GD and the one on 21 occurred after GD. For chromosome 11, most kataegis SNVs had low MAFs (black circles in figure below) supporting a kataegis after GD. However, a group of kataegis SNVs had slightly higher MAFs (black arrow). These SNVs were near a 7kb deletion. The deletion was called by SV breakpoints and visible on CNV profile (black arrows). These SNVs were likely from a separate kataegis event rather than being part of the chromothripsis. We did not discuss this possibility in the manuscript since it will not affect our main conclusion that kataegis can occur multiple times in a tumor. We have revised the text in Results section.*

Timing of chromothripsis

How many chromothripsis events occur pre or post genome doubling (or subclonally)? A simple overview/table could be instructive. Similarly for kataegis.

*Response: We have updated Tables S4 and S5 to provide such information.*

Timelines

Figure 7 appears as if the onset of molecular time coincides with the emergence of adenomas. This may be misleading as most likely the majority of point mutations will have occurred prior to neoplastic transformation (<http://dx.doi.org/10.1101/416800>).

*Response: We appreciate the comment and have revised Figure 7 to illustrate the uncertainty at adenoma stage.*

Minor issues

The author may want to consider some of the following issues which I stumbled across.

Germline SNV - usually called SNPs?

*Response: We have replaced the term “germline SNV” by “germline SNP” in the entire manuscript.*

Chromothripsis - the authors use the term very broadly, including BFBs, which I wouldn't have called chromothriptic.

*Response:* *The reviewer is correct to point out that we do use the term more broadly, given that chromoplexy, chromoanasynthesis, and some BFBs are difficult to distinguish from chromothripsis (our analysis of chromothripsis events in the 2700 genomes:* [*https://www.biorxiv.org/node/101741*](https://www.biorxiv.org/node/101741)*, in press at Nat Genet). We have modified to text to clarify this point.*

**Reviewer 2**

The manuscript is very nicely written and will be an important contribution to the field.

- It would be helpful to understand the selection of the 65 TCGA colorectal cancer subjects that had whole genome sequencing on tumor. While MSI frequency seems representative of most populations, the frequency of POLE exonuclease domain mutations seems a little high. If possible, it would be useful to further define tumor site by proximal/distal colon in the figures/tables.

*Response:* *Samples were selected for WGS to represent all gender and tumor stages to the extent possible. However, the reviewer is absolutely correct in that POLE mutant tumors are over-represented. Indeed, all POLE mutants from the entire TCGA colorectal cancer cohort (n=633) were subjected to WGS because they were of special interest to some of the authors—Dr. David Wheeler was the co-PI of the TCGA Tumor Genome Characterization Center at Baylor, where all the samples were sequenced, and he has published a study focusing on these tumors (Shinbrot et al. 2014). Tumor location and survival status were added to Table S1; unfortunately, we did not have enough samples to draw strong conclusion between tumor location (which span several categories) and genomic characteristics, and thus did not discuss this in the main text.*

With regard to genome duplications (GDs), it is not clear why the authors chose a threshold of more than half the genome showing duplication at the same time. It would be informative to see how much variability there is in GD designation as this threshold changes and to understand why half was chosen.

*Response:**The reviewer has raised a very good point. We have now plotted the number of co-amplified chromosomes in Figure S7b in all CIN tumors (pasted on the right). As you can see, the variation among the tumors is very large, and defining GD as having more than half of the chromosomes duplicated is admittedly somewhat arbitrary. However, we do need to define a threshold and we thought half was a reasonable choice given the plot above. A minor note: we had mistakenly characterized crc3913 as GD (10.5 chromosomes duplicated) but this has been changed to non-GD now.*

In the discussion, the authors talk about an enhanced genetic model, but it seems premature to state with certainty what occurs in this model for adenomas without studying adenomas or advanced adenomas.

*Response: We fully agree that our study does not provide any information about early stage adenomas and advanced stage adenomas. We have added the following in the Discussion section “Note that all tumors in our cohort are carcinomas. The order of genetic alterations in adenoma phase is adopted from the classical genetic model (Fearon and Vogelstein, 1990)”. We also revised the Figure 7 to indicate the uncertainty of adenoma stage.*

It would be useful if in the discussion the authors addressed the MSI positive case with CIN (crcA01R).

*Response: We have added the following texts to the paragraph where the crcA01R sample is mentioned: “Although CNVs were rare in MSI tumors (Figure 5) in general, their SV frequencies were comparable to those in CIN tumors (P=0.82, Wilcoxon rank sum test). We suspect that the ancestor cells of MSI tumors may have less chromosomal mis-segregation (thus no aneuploidy) but may have similar levels of erroneous* *DNA replication and error-prone DNA double strand break repair as CIN tumors.”*

Did the authors evaluate tumor stage or treatment in their analyses?

*Response: This is an interesting question. We had in fact tested the possible correlation of GD, tumor purity, clonal expansion time with tumor stage and overall survival in CIN tumors. However, most likely due to the small sample size, most tests were negative: tumor purity (P=0.12) and clonal expansion time (P=0.12) were not correlated with tumor stage (one-way ANOVA); GD was marginally under-represented in stage I tumors, but not statistically significant (P=0.32, chi-square test); and GD was not correlated with overall survival (P=0.32, likelihood ratio test by cox proportional hazards model). Tumor stage was significantly correlated with overall survival as expected (P=0.04, likelihood ratio test by cox proportional hazards model), but we do not mention it in the main text because this correlation is expected.*

- There appears to be a minor color error for Figure 2d

*Response: We thank the reviewer for his/her attention to the details! We assume that the reviewer is referring to the red/blue colors in the text “Clonal”. We interleaved red and blue on purpose, because the peak contains both red and blue SNVs (red for before, blue for after copy gain). We considered other ways to indicate this, and the mixed color was the best one we could come up with. Please note that this is explained in the figure legend: “In one-copy gain chromosomes (panel d), the SNVs in the middle peak of the MAF profile are SNVs present on one out of the three chromosomes and are a mixture of SNVs occurring both before (red SNVs) and after (blue SNVs) the copy gain, since red SNVs on the green chromosome are not duplicated.”*

- Did the authors use any orthogonal measures used to confirm SNV/indel calls?

*Response: The algorithms that we used are popular ones: MuTect, MuSE, and VarScan2. A great deal of validation has been done for each of these callers (e.g., Bian et al, Comparing the performance of selected variant callers using synthetic data and genome segmentation, BMC Bioinformatics, 2018; Bohnert et al, Comprehensive benchmarking of SNV callers for highly admixed tumor data, PLoS One, 2017). To further minimize variant calling error, we took those variants called by at least two algorithms. With such a stringent procedure, we think the variants we used are reliable.*

**Second round of review**

**Reviewer 3**

In this manuscript, Yang et al. performed comprehensive bioinformatic analysis to dissect the relative timing of somatic genetic alterations in 63 colorectal cancers with whole-genome sequencing data, and presented a refined tumor progression model which significantly expands our understanding of the tumorigenic process of human colorectal cancer. The paper is well-designed and well-written. In my opinion, the authors have already addressed all the reviewers’ comments, and it is appropriate to be published by Genome Biology.
